# Contemporary hybridization among *Arabis* floodplain species creates opportunities for adaptation

**DOI:** 10.1111/nph.70779

**Published:** 2025-12-10

**Authors:** Neda Rahnamae, Lukas Metzger, Lea Hördemann, Kevin Korfmann, Abdul Saboor Khan, Yasar Özoglan, Craig I. Dent, Samija Amar, Raúl Y. Wijfjes, Tahir Ali, Gregor Schmitz, Benjamin Stich, Aurelien Tellier, Juliette de Meaux

**Affiliations:** ^1^ Institute for Plant Sciences, Biocenter Cologne 50674 Germany; ^2^ Institute of Plant Ecology and Evolution, Heinrich‐Heine University Düsseldorf Düsseldorf 40225 Germany; ^3^ Professorship for Population Genetics, Department of Life Science Systems, Technical University of Munich Freising 85354 Germany; ^4^ Research Department for Limnology, Mondsee, University of Innsbruck Innsbruck 5310 Austria; ^5^ Institute of Ecology and Evolution, University of Oregon Eugene OR 97402 USA; ^6^ Department of Chromosome Biology, Max Planck Institute for Plant Breeding Research Cologne 50829 Germany; ^7^ CEPLAS: Cluster of Excellence on Plant Sciences, Heinrich‐Heine University Düsseldorf 40255 Germany; ^8^ Faculty of Biology, LMU Munich Planegg‐Martinsried 82152 Germany; ^9^ Julius Kühn Institute (JKI), Federal Research Centre for Cultivated Plants Institute for Breeding Research on Agricultural Crops Sanitz 18190 Germany

**Keywords:** *Arabis nemorensis*, *Arabis sagittate*, fine‐mapping, genetic incompatibility, hybridization, quantitative trait loci (QTL), segregation distortion

## Abstract

- Hybridization between closely related species is increasingly recognized as a major source of biodiversity. Yet, whether it can create advantageous trait combinations while purging harmful alleles remains unknown. To address this question, we studied *Arabis nemorensis* and *Arabis sagittata*, two endangered species that currently hybridize in a single hotspot.
- We chose two representative individuals originating from the hotspot, generated high‐quality annotated genome sequences, crossed them to form an F2 population, quantified segregation distortion along the genome, measured 22 phenotypic traits and mapped their genetic basis.
- Two genomic regions showed strong segregation distortion favoring *A. sagittata* alleles in the F2 and potentially accelerating their introgression. Fifty‐eight quantitative trait loci (QTLs) were identified for 20 traits, with additive and dominance effects best fitting Gaussian and logistic distributions, respectively. It was found that 48% of QTLs were unlinked to reduced fitness or segregation distortion. A major QTL affecting flowering time implicated Terminal-Flower 1 (*TFL1*) as a candidate gene for life‐history adaptation. QTLs did not overlap with recent selective sweeps, except for those controlling rosette size.
- Our findings offer unique insights into both the potential for adaptive trait combinations and the constraints imposed by hybrid fitness loss during incipient stages of hybridization.

## Introduction

Biodiversity is increasingly threatened by anthropogenic pressures and climate change, raising urgent questions about the mechanisms that allow species to persist (Moore & Hendry, 2009; Staudinger *et al*., 2012; Cochrane *et al*., 2016; Bontrager & Angert, 2019; Ceballos *et al*., 2020; Eichenberg *et al*., 2021; Cowie *et al*., 2022; IPCC, 2023; Schlaepfer & Lawler, 2023; Theissinger *et al*., 2023). One such mechanism is hybridization, the interbreeding of individuals from genetically distinct populations or species, which occurs frequently among close relatives (Blanckaert *et al*., 2023; Peñalba *et al*., 2024; Rosser *et al*., 2024). It can have both advantageous and detrimental consequences on the species receiving gene flow (Peñalba *et al*., 2024). First, interspecific hybridization can enable locally adapted alleles to be transferred across species barriers and thus enhance the adaptive potential of species (Seehausen, 2004; Pfennig *et al*., 2016; Abbott, 2017). Indeed, hybridization between populations with different ecological specializations can give rise to new, viable, and fertile hybrids equipped with novel trait combinations. Such combinations may improve the fitness of the population or even enable previously untapped habitats to be colonized (Buerkle *et al*., 2000; Rieseberg *et al*., 2003; Mallet, 2007; Abbott *et al*., 2013; Blanckaert *et al*., 2023). Hybridization can therefore have positive effects by providing a mechanism for fast evolutionary rescue (Becker *et al*., 2013; Todesco *et al*., 2020; Brauer *et al*., 2023; Nocchi *et al*., 2023).

However, the detrimental effects of hybridization are often more readily detected than its advantageous effects. Indeed, allelic incompatibilities can cause a massive fitness breakdown, when gene pools reunite after long periods of evolution in isolation (e.g. Wang *et al*., 2015; Cooper *et al*., 2018; Zuellig & Sweigart, 2018; Coughlan & Matute, 2020; Li *et al*., 2022; Moran *et al*., 2024). The overall fitness of the hybridizing population will be reduced if too many resources are used to produce poorly performing hybrids (Rhymer & Simberloff, 1996; Goulet *et al*., 2017). This phenomenon, sometimes described as ‘demographic swamping’, elevates the risk of extinction but may also select for allelic variations that reinforce species isolation (Hopkins, 2013; Goulet *et al*., 2017; Ma *et al*., 2019; Brauer *et al*., 2023).

Despite the interest in the positive consequences of hybridization and the abundant evidence for allelic incompatibilities (Bomblies & Weigel, 2007), we know little about how the positive and negative effects of hybridization interact, much less about how a genotype carrying adaptive alleles might emerge – especially against a background in which detrimental effects have been recombined out. Such ‘super genotypes’, although rare in the offspring of the first generation of hybrids (many of which may perform poorly), may represent new and exceptional combinations of adaptive alleles that, in selfing species, can determine the evolutionary success of hybridization. In addition, much focus has been placed on showing that introgressed alleles bring adaptive advantages to the recipient species (Edelman & Mallet, 2021). Yet, the role that natural selection has played in shaping the introgressing alleles of the donor species has seldom been examined. Addressing these two challenges is best achieved when hybridization can be studied as it proceeds, as this allows direct observation of the recombination and selection dynamics shaping introgression.

Here, we focused on a hybridization hotspot located along the banks of the Rhine River near Mainz, Germany, where two endangered *Arabis* species hybridize in a floodplain meadow (Dittberner *et al*., 2019, 2022). *Arabis nemorensis*, a perennial species within the Brassicaceae family (*Arabis hirsuta* tribe), inhabits floodplain meadows and is currently in a critical state in Central Europe, requiring special attention from conservation authorities (Schnittler & Günther, 1999; Burmeier *et al*., 2011). *Arabis nemorensis* is self‐pollinating and exhibits low levels of nucleotide diversity. Its endangered status is further intensified by its unique ecological requirements, and the loss of its natural habitat (Hölzel, 2005; Burmeier *et al*., 2011; Mathar *et al*., 2015; Dittberner *et al*., 2019). *Arabis sagittata*, another member of the same phylogenetic tribe (Karl & Koch, 2014), also perennial, is morphologically very similar but commonly found in calcareous grasslands and thus possibly more tolerant to drought (Dittberner *et al*., 2022). *Arabis sagittata* was recently observed in floodplains, where it naturally hybridizes with *A. nemorensis* (Dittberner *et al*., 2019, 2022). Both species mostly self in nature, although *A. sagittata* seems to experience rare outcrossing events more often (Dittberner *et al*., 2022).

Introgression analysis has revealed that gene flow between *A. nemorensis* and *A. sagittata* has happened in the past, but contemporary hybridization is restricted to the sympatric population, where intraspecific genetic variation is extremely low (Dittberner *et al*., 2019, 2022). Additionally, population genetics analyses estimated the divergence between *A. nemorensis* and *A. sagittata* occurred *c*. 900 000 generations ago, with practically complete isolation after the last glaciation (Dittberner *et al*., 2022).

Using the F2 progeny of a cross between two representative genotypes of *A. nemorensis* and *A. sagittata* from the hybridizing hotspot, we addressed the following questions: (1) what traits differ between species and how do these differences segregate in the F2 hybrids? (2) What is the genetic architecture of interspecific differences in this hybridizing hotspot? (3) Can a new combination of ecologically relevant traits arise and establish after hybridization? (4) Do quantitative trait locus (QTL) regions enriched in genomic regions carry footprints of past selection? Our study confirmed the extent of the genetic differences underpinning phenotypic divergence between two species. In addition, we found that some F2 hybrids show extreme trait values, despite their generally lower seed production compared to parental lines. Interestingly, incompatibility QTLs have a simple genetic basis, and some ecologically relevant QTLs are independent from the incompatibility QTLs. Collectively, these results indicate that a few offspring of hybridized species may potentially harbor a genotype with a combination of properties that none of the parents have.

## Materials and Methods

### Common garden experiment and phenotyping

To generate the hybrids, we crossed sympatric *Arabis nemorensis* genotype 10 with the *Arabis sagittata* genotype 69, collected from the banks of the Rhine River near Mainz in Riedstadt, Hessen, Germany, in 2015 and fully sequenced (Dittberner *et al*., 2019, 2022). Because nucleotide diversity within the population is very low (*A. nemorensis* π = 4.37e−5 and *A. sagittata* π = 1.32e−5, synonymous sites; Dittberner *et al*., 2022), we assume here that differences between these genotypes will predominantly reflect differences between species. Reciprocal F1s showed fitness comparable to parents (Supporting Information Fig. S1), and F2 progeny were raised under seminatural conditions in Cologne. In total, 1204 individuals (hybrids and parental replicates) were established. More than 20 morphological and life‐history traits, including growth, flowering time, leaf morphology, stem characteristics, fitness measures, and survival under a controlled submergence treatment, were recorded between November 2018 and April 2019. Full details of plant growth, experimental setup, and trait measurement protocols are provided in Methods S1.

### Phenotypic analyses

Phenotypic differences between the parental species were assessed using generalized linear models (Trait ~ Species + Tray) with false discovery rate (FDR) correction across traits. Effect sizes are reported as incidence rate ratios with 95% confidence intervals. To evaluate genetically based correlations within the F2 population, we used mixed models accounting for cross‐direction and tray effects, extracted residuals, and calculated pairwise Spearman correlations among corrected trait values. Significant correlations (α = 0.05) were used to construct a trait network, in which nodes represent traits and edges reflect positive or negative correlations. Full details of statistical models, software, and visualization procedures are provided in Methods S2 and Notes S1.

### DNA extraction and RAD‐seq library construction

We extracted DNA from frozen leaf tissue using the Nucleospin® 8 Plant II protocol. Restriction‐site‐associated DNA (RAD‐seq) libraries were prepared following Dittberner *et al*. (2019), with 801 F2 individuals sequenced across four NovaSeq runs at the Cologne Center for Genomics. Sequencing produced between 3 and 6 million reads per individual, covering *c*. 2% of the 248 Mb genome. Further details of library preparation and sequencing are provided in the Methods S3.

### Genome assembly, SNP calling, and linkage map construction

Chromosome‐scale genome assemblies for both parental lines were generated using PacBio HiFi and Hi‐C sequencing, scaffolded with reference‐ and linkage‐map–based approaches (ENA Project ID: PRJEB89863). Variant calling from RAD‐seq data and genetic map construction were performed using standard filtering and imputation procedures, resulting in 2082 single‐nucleotide polymorphism (SNP) markers across 742 F2 individuals. Full protocols, filtering thresholds, and software parameters are available in Methods S4 and S5, and Notes S2–S4.

### Segregation distortion and selection coefficient

We assessed segregation distortion within each region by applying a test of segregation distortion (profileMark) for each marker with Bonferroni correction using the ASMap package in R. To assess deviations from the Hardy–Weinberg equilibrium (HWE) and estimate selection coefficients, allele frequencies for each genotype (NN, NS, SS) were calculated based on the total number of individuals (*n* = 742). The observed genotype counts were used to compute allele frequencies (${\text{Freq}}_{N}$ and ${\text{Freq}}_{S}$) by summing homozygous and heterozygous contributions. Expected genotype frequencies under the HWE were then computed as ${\text{Freq}}_{N}^{2}$ (NN), 2 × ${\text{Freq}}_{N}$ × ${\text{Freq}}_{S}$ (NS), and ${\text{Freq}}_{S}^{2}$ (SS). These frequencies were scaled to expected counts by multiplying by the total number of individuals (Notes S4).

To determine significant deviations from the HWE, a chi‐squared goodness‐of‐fit test was performed for each locus. The observed and expected genotype counts were compared using the chi‐squared statistic, with 2 degrees of freedom. Significant deviations were identified based on a Bonferroni‐corrected *P*‐value threshold, accounting for multiple testing across all loci. The Bonferroni correction was applied by adjusting the significance threshold to 0.05 divided by the total number of markers (2082); this adjustment ensured that false positives were stringently controlled. Additionally, selection coefficients (*t* and *s*) were estimated by calculating the relative differences in ratios of observed to expected frequencies among genotypes. Specifically, the coefficients were defined $s_{\mathrm{NN}}=1-\frac{{\text{ratio}}_{\mathrm{NN}}}{{\text{ratio}}_{\mathrm{NS}}}$ and $s_{\mathrm{SS}}=1-\frac{{\text{ratio}}_{\mathrm{SS}}}{{\text{ratio}}_{\mathrm{NS}}}$, reflecting the relative fitness difference between homozygotes and heterozygotes (Carey & Ganders, 1980) (Notes S4).

Significance of genotype deviations from the HWE was assessed using a chi‐squared test for each marker. *P*‐values were adjusted for multiple testing using the FDR correction method, controlling the FDR at 5%. Markers with FDR‐adjusted *P*‐values below 0.05 were considered significant and were visually highlighted in the selection coefficient plot. Red and blue circles denote significant selection against NN (*t*) and SS (*s*), respectively. When both t and s were significantly greater than zero, markers were interpreted as showing potential selection favoring the heterozygote (NS).

### QTL mapping

QTL analyses were conducted using the r/qtl and qtltools packages (v.1.66; Broman *et al*., 2003; v.1.3.1; Delaneau *et al*., 2017) in R. QTL mapping was performed on the residuals of the phenotypic models described above (Phenotypic Analyses), in which we had already controlled for cross‐direction (cytoplasmic effects) and TrayBlock structure (positional effects). These residuals were then used directly in genome‐wide QTL scans for 22 traits listed in Table 1. However, survival after flooding, being a binomial trait, lacked a sufficient number of F2 individuals for QTL mapping (Notes S5).

**Table 1:** Differences in phenotypic traits between parents.

Trait	Mean *Arabis nemorensis*	Mean *Arabis sagittata*	Distance	*P*‐value
Days to Bolting (BT) (d)	157.2857	169.2857	12	7.67733569186116e−29
Days to Flowering (FT) (d)	183.5714	193.4286	9.8571	9.92819572840268e−26
Fertility Score (WS) (g)	0.0298	0.0295	0.0004	0.735982604
Inflorescence Height (PH) (cm)	35.2500	34.5714	0.6786	0.121159231
Lamina Length (Lam) (cm)	1.2729	1.4646	0.1916	0.011923973
Lamina L : W (LLW) (ratio)	1.4760	1.7938	0.3178	3.3370726452435e−10
Leaf Length (LL) (cm)	1.5562	1.7161	0.1599	0.787509948
Leaf Width (LW) (cm)	0.8589	0.8213	0.0376	0.103685956
No. of Stem Leaves (NL)	28.1429	18.1429	10	2.24983890109023e−33
Petal Length (Pet) (mm)	4.0569	5.3579	1.3010	6.04691680241348e−34
Petiole Length (Pti) (cm)	0.2833	0.2516	0.0317	0.002181075
Rosette Diameter 1 (RD1) (cm)	1.8331	2.2419	0.4087	0.713598816
Rosette Diameter 2 (RD2) (cm)	3.2946	3.6893	0.3947	0.081329783
Rosette Diameter 3 (RD3) (cm)	3.3611	3.4424	0.0813	0.072384866
Rosette Diameter 4 (RD4) (cm)	3.8805	3.9110	0.0305	0.111109462
Side Shoots (Ssh) (number)	2.7857	2.1429	0.6429	0.205905044
Stem Height (SH) (cm)	40.4286	34.4286	6	3.02830254946091e−07
Stem Leaf Density (SLD) (ratio)	0.6979	0.5271	0.1707	3.18884502716871e−20
Stem Leaf Length (SLL) (cm)	2.3894	2.7921	0.4028	0.004243421
Stem Leaf Width (SLW) (cm)	1.4021	1.2337	0.1684	0.014377018
Petiole L : Lamina L (PL) (ratio)	0.2464	0.1886	0.0579	0.001013407
Ground Shoots (Gsh) (number)	0.2143	1.2857	1.0714	3.27306200327204e−05

This table summarizes the phenotypic traits measured in the common garden experiment for F2 hybrids and their parental replicates (*A. nemorensis* and *A. sagittata*). Means represent the average value for each trait within parental replicates (35 individuals per species). ‘Distance’ shows the absolute difference between the species' means. The *P*‐values indicate the significance of the difference between species based on generalized linear models results.

For each trait, we used the *scantwo* function to perform a two‐dimensional genome scan with a two‐QTL model, applying the Haley–Knott regression algorithm (Haley & Knott, 1992). Penalties were calculated from 1000 permutations of the *scantwo* function to support the stepwise fitting of multiple QTL models. The *stepwiseqtl* function was then employed, with a maximum of five QTL, using Haley–Knott regression to search for optimized models. Allowing for more QTL did not change the results. We activated the *refine.locations* option in *stepwiseqtl* to improve the localization of QTLs and deactivated the *additive.only* option to allow for potential interactions between QTLs in the model.

We then looked into the summary output of *stepwiseqtl* to obtain information on the percentage of variance explained by each QTL, as well as the peak Logarithm of the odds (LOD) scores, and the additive (a) and dominance (d) effects for each significant QTL, reported relative to the *A. nemorensis* (N) allele (coded as the baseline parental allele in the QTL analysis), identified in the best stepwise models for each trait. For each identified QTL, we determined the 1.5 LOD confidence interval using the *lodint* function in r/qtl. Finally, we used the *segmentsOnMap* function from qtltools to visualize QTL segments on the genetic map, and ggplot2 (v.3.5.1; Wickham, 2011) to plot QTL effect sizes using the LOD scores obtained from the summary output of *stepwiseqtl*. To assess the distribution of estimated additive and dominance effects across traits, we performed the Shapiro–Wilk normality test in R (Villasenor Alva & Estrada, 2009) using standardized phenotype residuals derived from a GLM that accounted for cytoplasmic and positional effects, enabling comparisons across traits (Notes S5). Because QTLs associated with fertility or segregation distortion can inflate effect size estimates, we excluded those loci from this analysis. Specifically, we removed QTLs located in known segregation distortion regions (Chromosomes 4 and 7) and QTLs whose intervals overlapped with fertility score QTLs on the same chromosome. This allowed us to focus on the distribution of effect sizes among QTLs associated with other phenotypic traits.

### Analysis of whole‐genome resequencing data

We reanalyzed published whole‐genome resequencing data for 37 *A. nemorensis* and *A*. *sagittata* individuals, with *A. androsacea* as an outgroup (Dittberner *et al*., 2022). Reads were mapped to our new *A. nemorensis* reference genome, and SNP calling and filtering were performed using standard pipelines. Full details of read processing, variant calling, and filtering thresholds are provided in the Methods S6.

### Genome‐wide selection scans

Using the 37 full genome samples, we identified selective sweeps using biallelic SNPs and the omegaplus software (Alachiotis *et al*., 2012). The OmegaPlus statistics (omega) were calculated using a grid size of 200 000 bp. We defined a minimum window size (minwin) of 50 kb and a maximum window size (maxwin) of 100 kb for computing LD values between SNPs. Outlier omega statistics, which indicated selective sweeps had occurred, were determined based on the genome‐wide distribution of values. To minimize false positives arising from demographic processes, the cutoff values for the omega statistics were established using forward simulations in SLiM4 (Haller *et al*., 2019; Haller & Messer, 2023) that were simulated under the demographic history inferred by Dittberner *et al*. (2022). In short, the demography consists of two pulses of interspecific gene flow between both species, one ancient pulse that occurred directly after the species split and one recent migration event that occurred only between the sympatric populations.

We generated 10 000 neutral datasets of 2 Mb each under the demographic history of ancient and recent migration events that occurred between the two species, assuming a fixed recombination rate for each simulated block (5.5e−8). The maximum omega value from each simulated dataset was extracted, yielding a distribution of 10 000 maximum values. The 99th percentile of this distribution that was used as the threshold to identify outlier windows indicated selective sweeps had occurred.

To optimize sweep detection, we tested multiple combinations of grid, minwin, and maxwin parameters (from 50 to 200 kb for grid size, and 50 to 100 kb for minwin and maxwin). We then applied the optimized parameters to the real dataset of *A. nemorensis* and *A. sagittata*, extracting only the sweep regions that exceeded the simulation‐based threshold, which were considered high‐confidence selective sweep regions.

We further investigated the potential association between selective sweeps and phenotypic traits by analyzing the overlap between the identified sweep regions and QTLs in *A. nemorensis* and *A. sagittate* (Notes S6). We used only high‐confidence selective sweeps in 200‐kb windows across the genome and overlaid these with the positions of all QTLs. We focused on the 10 percent quantile regions around the QTL peak positions to increase specificity.

### Fine‐mapping the largest effect QTL

To fine‐map the largest effect QTL, which was associated with the Days to Flowering trait (first QTL on Chromosome 8), we identified F2 lines that were heterozygous for the QTL of interest and homozygous for other Days to Flowering QTLs; a total of 15 lines resulted (Tables S1, S2). We sowed 90 F3 seeds per line (3 seeds per pot) in trays and transferred these to the cold chamber for vernalization. After 2 wk, we transplanted one seedling into each 7 × 7 cm pot filled with *Topferde* (Einheitserde, Sinntal‐Altengronau, Germany), resulting in 30 seedlings per line. Pots were kept in the glasshouse for 6 wk, after which plants were transplanted into larger 9 × 9 cm pots and moved to cold frames in the garden for 7 wk. During this period, leaf material was harvested from each plant, and DNA was extracted from fresh leaves using the NucleoSpin® 8 Plant II protocol. Subsequently, the plants were transplanted into new 11 × 11 cm pots and placed on tables in the garden under a bird‐protected cage. We recorded flowering time, inflorescence height, internode length, number of shoots, plant height, stem leaf density, stem height, and rosette diameter (RD). Two successive trials were carried out on 483 plants; the first started on 26 September 2022, the second on 16 November 2022. Plants flowered between April and June 2023.

geneious prime® (v.2024.0.5) was used to design multiple species‐specific primers targeting the QTL region based on the *A. nemorensis* and *A. sagittata* genomes. We designed five pairs of PCR markers, which divided the QTL region into four intervals. PCR was conducted on the DNA of 407 plants to identify recombinants and locate their recombination events within the predefined intervals. We built a quantitative model using the *glm* function from the stats package (v.3.6.2) in R, accounting for both family and trial effects as fixed factors (glm(flowering_time ~ family × (exp/tray_garden), family = quasipoisson)), in which trays were nested within experiments because tray labels were reused across experiments. We included the interaction term (family × (exp/tray_garden)) because families showed variation among experiments in flowering time. Flowering time was defined as the number of days from sowing to the first open flower (days after sowing). Then, we extracted residuals and ran another model on the residuals (glm(res_ftime ~ interval1 + interval2 + interval3 + interval4, family = gaussian)). The model was run for each interval, with the interval that best explained variation in flowering time residuals containing the flowering time QTL (Notes S7). The same procedure was then applied to assess the impact of each interval on traits with overlapping QTLs in the same region as the F2 mapping population (Inflorescence Height, RD, and Stem Height). Parental DNA was used as a positive control, and nuclease‐free water (no template) was included as a negative control to monitor for contamination and confirm that amplification was specific to DNA templates.

## Results

### Phenotypic differences of ecologically relevant traits

In total, 1193 individuals germinated in the glasshouse and were set to grow in a common garden situated at the University of Cologne. All replicates of *A. nemorensis* and *A. sagittata* survived the common garden experiment (35 *A. nemorensis* and 35 *A. sagittata*). *Arabis nemorensis* flowered *c*. 12 d earlier than *A. sagittata* (*P* < 0.001, Table 1). Mean values for 13 of the 22 scored traits differed between species (Table 1; Notes S1).

**Fig. 1:**
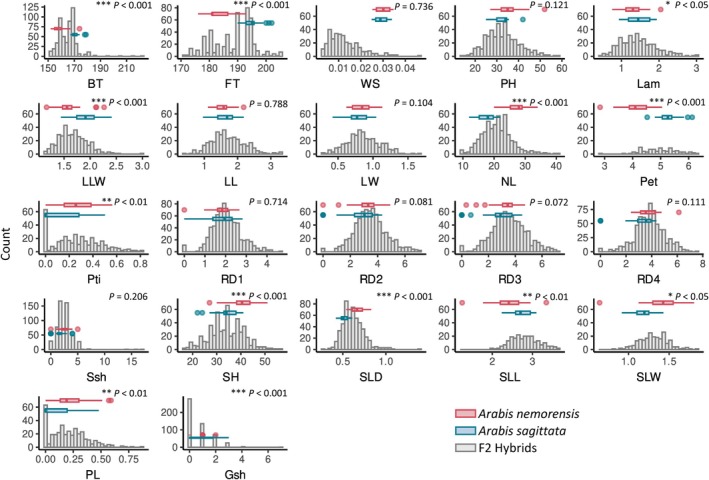
Phenotype distribution in *Arabis* F2 progeny and their parental lines. Histograms illustrate the distribution of traits in the 1193 plants of the F2 generation grown in a common garden. Boxplots highlight trait variations between the two parental lines and indications of transgressive segregation in their offspring. The box represents the interquartile range, the horizontal line shows the median, whiskers extend to 1.5x IQR, and points beyond the whiskers are plotted as outliers. The *P*‐values and stars depict the significance of trait differences between parents: *, *P* < 0.05; **, *P* < 0.01; ***, *P* < 0.001. They were obtained with generalized linear models (Trait ~ Parent + Tray). Parental contrasts were obtained via emmeans and *P*‐value were corrected for false discovery rate. BT, Days to Bolting; FT, Days to Flowering; Gsh, Ground Shoots; LL, Leaf Length; LW, Leaf Width; Lam, Lamina Length; LLW, Lamina Length‐to‐Width Ratio; NL, Number of Stem Leaves; PH, Inflorescence Height; PL, Petiole Length‐to‐Lamina Length Ratio; Pet, Petal Length; Pti, Petiole Length; RD1‐4, RD at four time points; SH, Stem Height; SLD, Stem Leaf Density; SLL, Stem Leaf Length; SLW, Stem Leaf Width; Ssh, Side Shoots; WS, Fertility Score – Seed Production.

*Arabis nemorensis* individuals displayed a markedly higher number of stem leaves compared to *A. sagittata* (mean difference = 10 leaves, *P* < 0.001), as expected for a trait that is often used to determine the taxonomy of these species (Titz, 1979). The RD of the parental genotypes did not differ significantly after 1, 2, 3 and 4 wk of growth (all *P* > 0.05). Moreover, trait variance among F2 hybrids was generally larger than trait variance between their parental genotypes, suggesting transgressive segregation (Fig. 1). Although the seed production of parents did not differ significantly (Fertility Score or WS, *P* = 0.735982604), the low fertility observed for most F2 contrasted with the normal fertility of F1s (Fig. S1) and indicated outbreeding depression (Fig. 1).

After 4 wk of flooding, the survival rates of parental genotypes showed no significant differences ($\chi^{2}$(1, *n* = 14) = 0.43, *P* = 0.5116).

The cross‐direction impacted several traits, suggesting maternal influence and potential cytoplasmic effects (Table S3). Traits such as Stem Leaf Length (SLL, *P* = 3 × 10^−5^), RD at early stages (RD1, *P* = 8 × 10^−5^), and Petiole Length (Pti, *P* = 0.006) exhibited strong associations with the direction of the cross. Additionally, Petiole Length‐to‐Lamina Length Ratio (PL, *P* = 0.011), RD 2 (RD2, *P* = 0.013), and RD 3 (RD3, *P* = 0.03) also displayed significant maternal effects. In terms of direction, *A. sagittata* maternal cytoplasmic inheritance increased SLL (consistent with the larger parental SLL of *A. sagittata*), but decreased RDs RD1‐RD3 (opposite to the parental difference, since *A. sagittata* parents had larger rosettes). For Pti and PL, in which *A. nemorensis* parents were larger, having *A. sagittata* as the mother increased trait values, again opposite to the parental difference. *Arabis sagittata* maternal cytoplasmic inheritance decreased rosette lamina size on the rosette but increased it on the stem (Table S3; Fig. S2). Thus, maternal effects were trait‐specific, sometimes reinforcing and sometimes opposing parental trait differences. By contrast, the majority of traits, including Days to Bolting (BT), Days to Flowering (FT), Fertility Score (Seed Production or WS), and structural traits such as Inflorescence Height (PH) and Number of Stem Leaves (NL), showed no cytotype effects (*P* > 0.05), indicating limited or negligible maternal influence.

Spearman correlation analysis of 22 traits in the F2 population revealed relationships among traits (Figs S3, S4). Traits associated with leaf shape, such as Lamina Length (Lam) and Leaf Width (LW), exhibited strong positive correlations (*r* > 0.8, *P* < 0.001). Similarly, RD (RD1–RD4) measured at different time points showed strong positive correlations (*r* > 0.7, *P* < 0.001).

Developmental traits, such as Days to Bolting (BT) and Days to Flowering (FT), showed moderate correlations with vertical growth traits, including Stem Height (SH) and Inflorescence Height (PH). For instance, Inflorescence Height was positively correlated with Days to Flowering (*r* = 0.535, *P* < 0.001), suggesting that later‐flowering individuals allocate more resources to vertical growth. By contrast, Fertility Score (Seed Production or WS), a component of fitness, exhibited weaker and often insignificant correlations with other traits, indicating potential independence from vegetative and structural phenotypes.

Petiole traits, including Petiole Length (Pti) and its ratio to Lamina Length (PL), were moderately correlated with rosette size and leaf shape traits such as Leaf Width (LW). Overall, these findings highlight the interdependencies among structural traits (e.g. plant size, leaf shape), developmental traits (e.g. flowering time, rosette size over time), and fitness components in *Arabis* F2 hybrids. The statistical analyses of ecologically relevant traits in the common garden experiment provide valuable insights into the extent of phenotypic integration and the nature of resource allocation strategies in the F2 hybrid population.

### Genetic map, pre‐ and postzygotic selection distortion

To investigate the genetic architecture underlying trait variation, we used a reduced sequencing approach to determine the genotypes of 742 F2 individuals at 2082 reliable SNP markers with the help of high‐quality genome assembly (Fig. S5; Notes S2), and constructed a genetic map. The map consisted of eight linkage groups (LGs), which were numbered according to the chromosome numbers of *Arabis alpina*. The length of the linkage map was 240 cM, with 160–369 markers per chromosome (Table S4). Contrasting the genetic and physical distances of SNPs along chromosomes showed that recombination was higher on chromosome arms compared to in centromeric regions (Figs S6, S7).

**Fig. 2:**
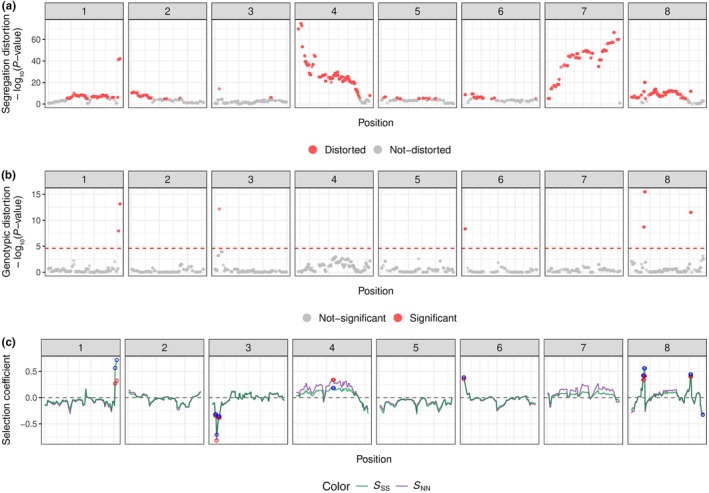
Segregation distortion, genotypic distortion and strength of selection along the genome in *Arabis* F2 progeny. This figure illustrates segregation distortion, genotypic distortion, and the strength of selection across the genome in the F2 population. The *x*‐axis represents marker positions along the genome across the eight chromosomes (2082 markers). The *y*‐axis in (a, b) shows −log_10_ (*P*‐value); the *y*‐axis in (c) represents the selection coefficient. (a) Gametic distortion: shown by the deviation of single‐nucleotide polymorphism from expected Mendelian segregation ratios, assessed using a segregation distortion test (profileMark) with Bonferroni correction for multiple testing (threshold *P* < 2.4 × 10^−5^). Significant deviations are highlighted in red. A total of 1257 markers are significantly distorted. (b) Genotypic distortion: calculated as the deviation of observed genotypes from expected values according to the Hardy–Weinberg equilibrium (HWE), assessed using a chi‐squared test. Bonferroni‐adjusted significant differences are highlighted in red. Only 47 markers surpassed the Bonferroni significance threshold. (c) Selection coefficient (*s*): calculated based on deviations from expected allele frequencies, with selection on allele *S* represented in green and on allele N in purple. Red and blue circles indicate markers where genotype frequencies significantly deviate from the HWE (false discovery rate (FDR)‐adjusted *P* < 0.05, chi‐squared test), suggesting selection at those loci. Red circles represent significant selection against the NN genotype (*t*); blue circles represent selection against the SS genotype (*s*). Loci where both *t* and s are significant and positive, indicate potential selection favoring the heterozygote (NS).

The analysis of allele and genotype frequencies along the genome uncovered strong segregation distortions in the F2 population. A strong segregation distortion was found on Chromosomes 4 and 7 with the percentage of N alleles dropping from 50% (expected) to 32% (Fig. 2a). Because the sequencing data were mapped on the high‐quality *A. nemorensis* genome, we can conclude that this depletion is clearly not due to mapping biases. To disentangle prezygotic from postzygotic mechanisms of allele distortion, we estimated the difference between expected and observed genotype distributions across the genome using a chi‐squared test (Fig. 2b), and inferred selection coefficients against each type of homozygote. This analysis showed that the segregation distortions on Chromosome 7 and on the tip of Chromosome 4 were not due to postzygotic selection on the genotype. Yet, it identified six regions in the genome with a significant excess of heterozygotes, three of which were particularly strong on Chromosomes 1 and 8. It further highlighted one region in Chromosome 3 with massively depleted homozygotes.

The extent of segregation distortion found throughout the genome appeared to be mostly due to interactions between alleles and not between different loci. For example, no genetic association was observed between the distortion on Chromosomes 4 and 7 (Fig. S8). Nevertheless, we find biased transmissions of parental alleles on Chromosomes 2 and 5, as well as Chromosomes 3 and 8 (Fig. S8).

### Genetic architecture of ecologically relevant traits

**Fig. 3:**
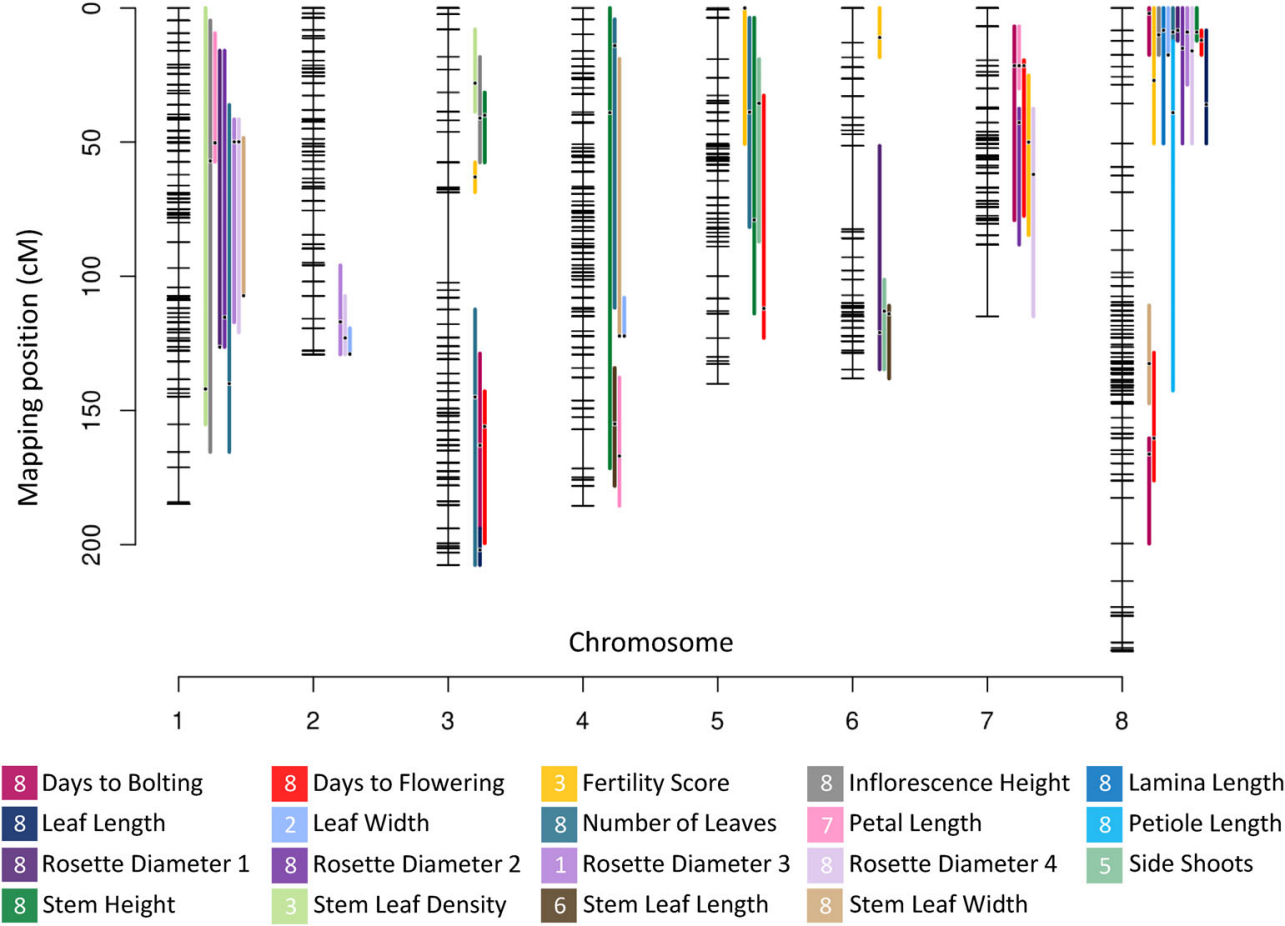
Genetic map with quantitative trait loci (QTLs) for ecologically relevant traits in *Arabis* F2 progeny. The plot illustrates significant QTLs that were detected and located on the genetic map. Horizontal bars represent mapped single‐nucleotide polymorphism (SNP) markers. Gaps between bars stand for the genetic distance between SNP markers in cM (centimorgan). Traits are listed in alphabetical order. The chromosome containing the strongest QTL of each trait appears in the square.

We detected significant QTLs for 20 of the 22 traits we scored (Table 2). The number of QTLs per trait ranged from 1 (Lamina Length) to 5 (Days to Flowering). The most significant QTL (LOD score > 40) was identified on Chromosome 8, accounting for more than 20% of the variation in flowering time. By contrast, the lowest LOD score (3.027) was observed in one of the Leaf Width QTLs. In total, 58 QTLs were identified along the genome (Table 2). We observed overlapping QTLs for multiple traits, including 9 on Chromosome 1; 3 on Chromosome 2; 11 on Chromosome 3; 4 on Chromosome 4; 3 on Chromosome 5; 2 on Chromosome 6; 6 on Chromosome 7; and 20 on Chromosome 8 (Fig. 3). Notably, one QTL associated with the Fertility Score on Chromosome 6 did not overlap with any other QTLs (Fig. 3; Table 2).

**Table 2:** Quantitative trait loci (QTLs) of ecologically relevant traits in the *Arabis* mapping population.

Trait	No. of observations	Significance of parental differences	Chr	Position (bp)	Position(cM)	LOD	%Variance explained	Est *a*	Est *d*
Days to Bolting (BT)	631	***	3	20 040 893	163	5.146	3.130	0.005914	−0.012765
			7	1835 566	21.5	5.359	3.263	0.010990	0.002648
			8	2061 023	12.2	7.786	4.783	−0.011989	0.000447
			8	19 500 950	134.2	12.396	7.746	0.016244	−0.003620
Days to Flowering (FT)	578	***	3	26 882 840	190	9.915	4.785	0.012242	−0.001815
			5	23 418 119	111	4.868	2.289	−0.007092	0.003427
			7	1835 566	21.5	11.241	5.423	0.011243	−0.001854
			8	2061 023	13	42.112	23.075	−0.021252	0.008320
			8	18 832 553	133	9.374	4.488	0.009159	−0.005437
Fertility Score (WS)	552		3	8362 080	63	8.774	6.165	−0.056270	−0.366350
			6	1052 484	10	3.897	2.683	0.172390	−0.055580
			7	3192 272	31	6.663	4.641	0.205690	−0.053080
			8	3118 527	17	5.070	3.507	0.178080	−0.028820
Inflorescence Height (PH)	578	.	1	9004 320	49.9	4.166	2.385	−0.012682	0.062053
			3	2664 384	27	11.394	6.714	0.078301	0.016361
			8	1180 103	6	20.789	12.728	−0.094477	0.076771
			8	10 128 705	101	6.383	3.686	−0.062798	−0.038649
Lamina Length (Lam)	436	*	8	5481 058	36.0	4.334	4.475	−0.075590	0.042250
Lamina L : W (LLW)	436	***	3	6744 303	61	15.635	14.890	0.089464	−0.031374
			4	38 897 287	163	4.045	3.620	0.041307	0.028487
Leaf Length (LL)	436		1	13 071 968	72.4	4.458	3.766	0.071867	−0.012244
			3	14 255 522	136.2	5.214	4.423	−0.007829	0.118366
			3	29 031 007	201.4	5.617	4.775	0.066584	−0.094772
			7	14 342 773	68.8	4.284	3.615	−0.020747	0.086727
			8	5624 776	35	9.433	8.183	−0.104748	0.036218
Leaf Width (LW)	436		1	21 722 429	116	4.313	3.893	0.069770	0.045460
			2	19 435 694	87	3.027	2.713	−0.043950	0.056000
			2	25 576 894	129	5.987	5.452	0.091250	0.012400
			8	4356 973	32	6.705	6.129	−0.081970	0.036770
Number of Stem Leaves (NL)	625	***	3	14 075 550	130.9	4.581	2.557	0.025147	0.059693
			4	7558 305	18	4.001	2.228	0.056356	0.010451
			5	16 624 270	70.7	5.384	3.014	−0.055649	0.004690
			8	1180 103	7	28.109	17.139	−0.119377	0.052082
Petal Length (Pet)	106	***	1	9074 627	51	4.863	15.820	0.064910	0.039080
			7	1835 566	21.5	5.689	18.850	0.079160	0.013480
Petiole Length (Pti)	361	**	8	10 723 493	107.5	3.814	4.749	−0.153200	−0.035130
Rosette Diameter 1 (RD1)	695		1	17 879 753	95	4.498	2.747	0.068440	−0.020590
			3	623 617	2.3	3.632	2.212	0.054350	0.023100
			8	4362 085	28	6.942	4.275	−0.074250	0.032990
Rosette Diameter 2 (RD2)	700	.	1	21 954 996	115	6.158	3.703	0.079730	0.013670
			7	5769 578	42.7	3.839	2.291	−0.056060	0.024000
			8	4356 973	31	7.822	4.729	−0.085450	0.033910
Rosette Diameter 3 (RD3)	696	.	1	9004 320	49.9	9.389	5.683	0.102710	−0.005208
			2	23 541 930	118	4.024	2.392	0.043216	0.071859
			8	2718 077	18	7.209	4.332	−0.087105	0.022622
Rosette Diameter 4 (RD4)	701	*	1	21 722 429	115.3	4.330	2.678	0.061815	0.014845
			8	4356 973	32	7.814	4.888	−0.078677	0.035138
Side Shoots (Ssh)	595	*	4	36 501 835	137.7	3.859	2.842	−0.119930	−0.038550
			5	5129 643	35.5	4.875	3.605	0.126580	0.031450
Stem Height (SH)	625	***	1	29 847 516	162	3.594	1.778	−0.029247	0.036282
			3	4035 375	36	13.598	6.984	0.074366	0.005981
			4	38 897 287	160	4.386	2.177	0.042699	−0.004189
			8	1180 103	6	26.851	14.500	−0.091994	0.070533
			8	10 506 892	102	7.858	3.951	−0.059677	−0.004408
Stem Leaf Density (SLD)	624	***	3	3491 752	29	5.760	4.162	−0.052426	−0.018081
Stem Leaf Length (SLL)	259	**	6	21 070 306	134.8	5.918	9.356	0.046349	0.011155
			8	3118 527	15	3.967	6.163	−0.035380	0.011240
Stem Leaf Width (SLW)	259	***	8	18 832 576	132.5	13.228	20.958	−0.084603	0.017725

This table summarizes the number, chromosomes, peak positions in base pairs (bp) and centimorgan (cM), LOD scores, percentages of phenotypic variance explained, and estimated additive and dominance effects of allele *S* for each QTL. The number of observations per trait in the F2 population is indicated. Significance differences between parental lines are denoted as follows: ***, *P* < 0.001; **, 0.001 < *p* < 0.01; *, 0.01 < *p* < 0.05; ., 0.05 < *P* < 0.1 (Supporting Information Notes S5).

The largest effect QTLs for traits such as Days to Bolting, Days to Flowering, Inflorescence Height, Leaf Length and Width, Number of Stem Leaves, and RD after 1, 2, and 4 wk, as well as Stem Height and Stem Leaf Width, were all located on Chromosome 8. Additionally, the strongest QTLs for Petal Length, RD 3, Side Shoots, Stem Leaf Density, and SLL were identified on Chromosomes 7, 1, 5, 3, and 6, respectively. For all traits, the additive effect of the *A. nemorensis* allele was either positive or negative, confirming that transgressive genetic variation can arise in most traits in this selfing species by fixing a new combination of alleles (Table 2).

The distribution of fertility scores, which was measured as the weight of seeds contained in 10 siliques, indicated that F2 individuals tended to be less fertile than the parental lineages. The genetic architecture of fertility score variation was dominated by a large‐effect QTL (LOD = 8.774) on Chromosome 3 (Fig. S9), which explained > 30% of the phenotypic variation and involved interallelic incompatibility at position 8 362 080 bp on Chromosome 3 (QTL interval: 6 744 303 to 9 601 991 bp; Fig. S9). Individuals with the NS heterozygous genotype at this marker displayed markedly lower fertility. In addition, three smaller QTLs explaining 2.683%, 4.641%, and 3.507% of the variation were found on Chromosomes 6, 7, and 8, respectively. It can be concluded that the genetic basis of outbreeding depression is relatively simple in this population.

Of the 22 traits scored, Ground Shoots (Gsh), Petiole Length‐to‐Lamina Length ratio (PL), and Survival to Flooding revealed no significant QTL. For two of these three traits (Ground Shoots, Petiole Length‐to‐Lamina Length ratio), significant differences between species were observed. The absence of QTLs for these traits suggests they may have a polygenic genetic basis, and the effect of individual variants may be too small to be detected.

**Fig. 4:**
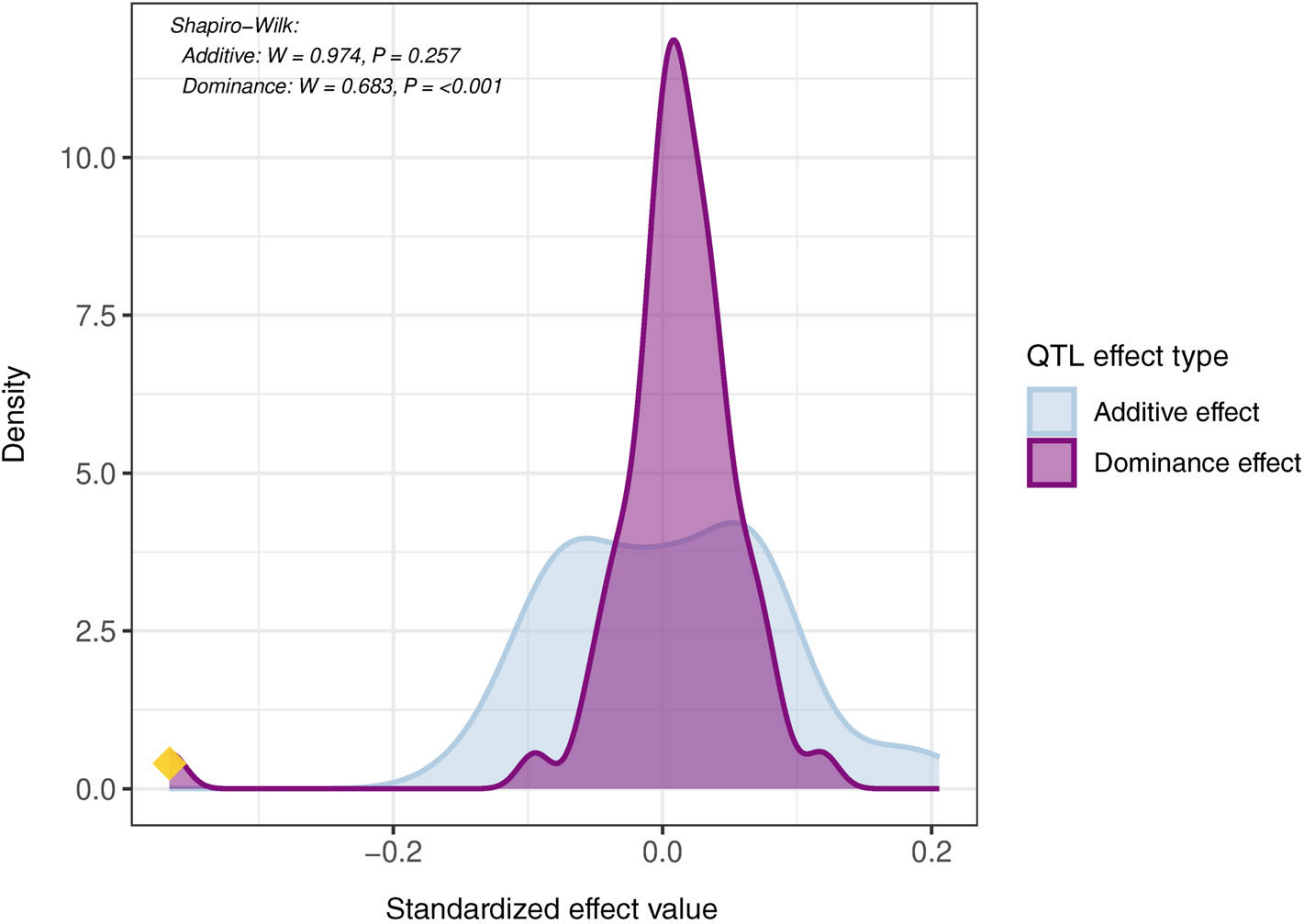
Distribution of standardized quantitative trait loci (QTL) estimated additive and dominance effects in *Arabis* F2 progeny. The figure displays the distribution of standardized estimated additive and dominance effects for QTLs associated with ecologically relevant traits identified in the F2 population. Each curve represents the density of standardized effect sizes for a distinct type of genetic effect: additive effects (in light blue) and dominance effects (in purple). The yellow diamond marks the dominance effect of the strongest QTL for Fertility Score (WS) on Chromosome 3. Effect values were estimated using interval mapping on standardized phenotype residuals derived from a GLM that accounted for random effects, enabling comparisons across traits. Density distributions illustrate the range and frequency of QTL contributions. Additive effects showed a Gaussian distribution (Shapiro–Wilk: *W* = 0.974, *P* = 0.257), whereas dominance effects approximated a logistic distribution after excluding the outlier dominance effect of a QTL on Chromosome 3 (Shapiro–Wilk: *W* = 0.683, *P* < 0.001).

We conclude that the two parental lineages differ genetically in many traits, with effects of various sizes. The number of detected QTLs further allowed us to describe the effect sizes of genetic variation that can be generated by hybridization in this system. The standardized additive effects were normally distributed (Shapiro–Wilk: *W* = 0.9744, *P* = 0.2573; Fig. 4), ranging from a minimum of −0.15 to a maximum of 0.20 with a median of 0.008 (mean = 0.005, SD = 0.08, skewness = 0.29, kurtosis = 2.6). QTLs with the largest effects controlled flowering time, stem height, inflorescence height, leaf number, and lamina shape contributing to the heavy tail of the distribution of additive effect (Figs 4, S10). Standardized dominance effects instead approximated a logistic distribution (Shapiro–Wilk: *W* = 0.683, *P* < 0.001), ranging from −0.36 to 0.11 with a median of 0.012 (mean = 0.008, SD = 0.062, skewness = −3.70, kurtosis = 23.5). The dominance effect of the fertility score QTL on Chromosome 3 was an outlier to this distribution (Fig. 4). Since it uses standardized phenotypic values, this distribution can help benchmark genetic variation in other studies of hybridization.

### Genes responsible for flowering time

The largest effect QTL detected in this study impacts the timing of flowering and is located on Chromosome 8, where two well‐known genes that control flowering time are found: *FLC*, a regulator involved in the vernalization pathway, and *CONSTANS*, a regulator involved in the photoperiod pathway (Andrés & Coupland, 2012). *FLC* has been shown to be a key variant shaping flowering time in many Brassicaceae species, including the congeneric species *A. alpina* (Albani *et al*., 2012). The gene *CONSTANS* is also located within the boundaries of this QTL. In order to test whether one of these candidate genes was responsible for the variation, we selected 15 F2 individuals that were heterozygous in the QTL region of Chromosome 8 and homozygous on the other QTLs, and we grew 30 of their seeds. A total of 410 plants were assessed for flowering time in 15 F3 families and two trials (Fig. S11, Tables S1, S2).

**Fig. 5:**
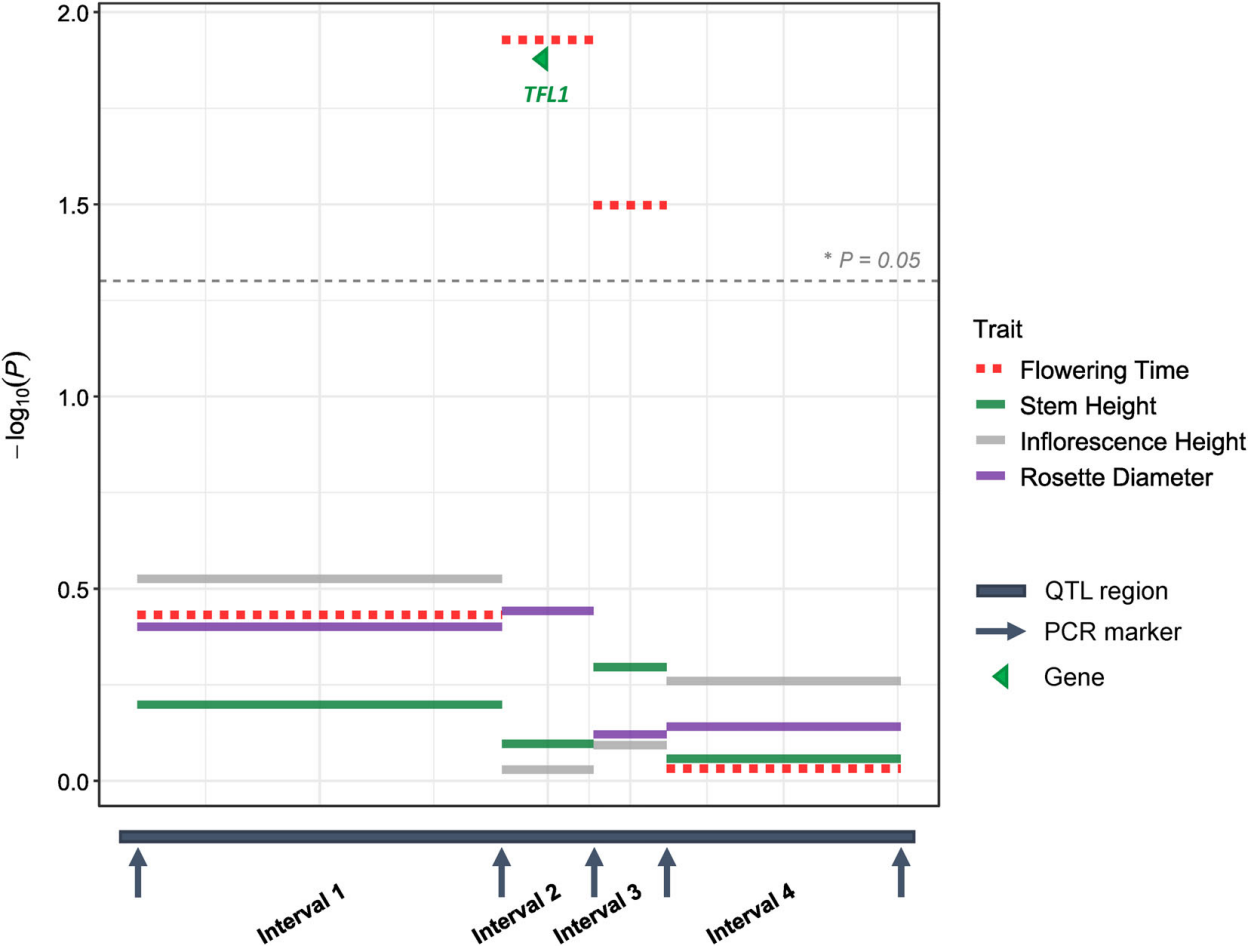
Association between four DNA fragments and flowering time within 15 *Arabis* F3 families segregated for the chr8 flowering time quantitative trait loci (QTL). The figure illustrates the role of genomic intervals within the QTL region in explaining variations in Flowering Time, Inflorescence Height, Rosette Diameter, and Stem Height. The *x*‐axis represents the region of the strongest Flowering Time QTL, divided into intervals defined by species‐specific primers with adjusted lengths for fine‐mapping. The dashed line indicates the significance threshold. Different bar colors represent different traits. The *TFL1* gene was detected in Interval 2, which shows the strongest effect on flowering time.

With 410 plants and a QTL region that was *c*. 17 cM, we expected 80 recombinants. Interestingly, we identified 138 recombinants, suggesting that the recombination rate had been slightly underestimated in the F2 population. We compared four models to identify the chromosomal fragments that best explained variation in flowering time, after accounting for all other factors of the experimental design. Using Akaike's criterion and comparing *P*‐values, we identified Fragment 2 as the most likely to contain the variants causing a difference in flowering time (Fig. 5, *P* = 0.0118). This segment ranges from position 1 831 324 bp to position 2 125 083 bp at the beginning of Chromosome 8. Although this *c*. 300 kb region does not contain *FLC* or *CO*, it contains 64 other genes, 31 of which have a known ortholog in *Arabidopsis thaliana*. Only one of these, Terminal‐Flower 1 (*TFL1*), is known to regulate flowering time in *A. thaliana* and *A. alpina*, with a loss of function inducing early flowering in both species (Cerise *et al*., 2023). *Arabis sagittata TFL1* differs from the *A. nemorensis* copy by two amino acid exchanges: Asparagine 3 is changed to Isoleucine and Serine 50 is changed to Tyrosine. The *A. nemorensis* version at both sites is conserved in most Brassicaceae, like *A. thaliana*, where *TFL1* function has been experimentally verified (Andrés & Coupland, 2012). Nevertheless, both positions are not strictly conserved.

In contrast to most *TFL1* genes from the Brassicales, several species within (*Capsella rubella*, *Sinapis alba*) and outside of this group are missing the start codon and protein translation starts at the conserved Methionine codon at Position 4. At Position 50, Serine is the most common amino acid not only in the Brassicales group but also in other plant groups; other amino acids (Alanine, Threonine, Phenylalanine) are found in other Brassicaceae *TFL1* sequences. Because *A. sagittata* carries derived amino acids at two moderately conserved positions, gene activity may be decreased compared to the more ancestral *A. nemorensis* allele. However, fine‐mapping allowed us to exclude the role of flowering loci such as *FLC* or *CO* that locate on the same chromosome arm of chromosome 8.

Although there were QTLs for Inflorescence Height, RD, and Stem Height, on Chromosome 8 in F2s, the chromosomal region that was fine mapped did not explain the variation of these traits in F3s. Inflorescence Height, RD, and Stem Height are thus controlled by QTL(s) independent of the QTL for Flowering Time in the *TFL1*‐containing fragment, with the S allele advancing flowering compared to the N allele.

### Overlap of parental QTLs with the history of selection in the parental lineages

In order to test whether genetic differences between lineages were influenced by natural selection in the two parental lineages, before hybridization, we used previously published population genomics data to determine regions with signatures of selective sweeps (Dittberner *et al*., 2022). We detected 35 and 82 genome regions carrying signatures of selective sweeps in *A. nemorensis* and *A. sagittata*, respectively, which prompted us to ask whether variable traits were disproportionately targeted by natural selection in their lineage or origin (Fig. S12). Many of the 58 QTL regions spanned large parts of chromosomes, sometimes overlapping with multiple independent selective sweeps (Fig. S13). We tested whether the distance between QTL peak and selective sweep was shorter than expected by a permutation test. QTL peaks did not tend to be located close to selective sweep signatures. We also did not detect significant overlaps between regions with distorted segregation and regions carrying selective sweeps (not shown). The genetic variation segregating in the F2 population thus does not appear to reflect the history of selection in the hybridizing lineages, yet some individual QTLs may reflect recent selection. For example, sweeps overlapped with five of the seven genomic regions with QTLs for rosette size (Fig. S12). By contrast, no signature of a putative selective sweep was found in the *TFL1* region.

## Discussion

Hybridization between species is pervasive in both past and contemporary ecosystems (Lewontin & Birch, 1966; Stull *et al*., 2023). By bringing together alleles that contribute to different ecological specialization, it can facilitate the emergence of a new allelic combination that may surpass the performance of the parental genotypes and be fixed via selfing. Hybridization may therefore become pivotal in the rescue of endangered selfing species with very low levels of genetic diversity (Frankham, 2015). Here, we take advantage of a hybridization hotspot we identified previously to disentangle the genomic and ecological properties of genetic variation released after hybridization; some of these properties may propagate in the population via selfing. An F2 generation was obtained by crossing individuals' representative of the two species in the sympatric population where they naturally hybridize (Dittberner *et al*., 2019, 2022).

The genetic analysis of allelic transmission in the F2 population first revealed the genomic barriers that skew allele segregation in hybrid offspring. Indeed, several regions of the genome displayed highly distorted transmission. For the two most strongly distorted regions on Chromosomes 4 and 7, the genotypic composition of the F2 fits Mendelian expectations, suggesting that the distortion happens before fertilization, as a result of biased gamete formation. Both of these distortions drive the *A. sagittata* allele to high frequencies in the F2 offspring population. We thus conclude that we observe meiotic drive in our cross, that is, the predominance of one allele among mature gametes produced in a heterozygous individual. Meiotic drive is a very strong evolutionary force (Sandler & Novitski, 1957; Pinkas *et al*., 1985; Clarke *et al*., 2004; Domínguez *et al*., 2005). It can massively accelerate the fixation of alleles linked to the driver locus. Driver alleles may outcompete nondriver alleles during pollen tube growth in heterozygotic selfing plants, thereby biasing the paternal transmission of alleles in favor of the offspring (e.g. Snow & Spira, 1996; Aronen *et al*., 2002; Lankinen *et al*., 2009). In female gametes, any molecular change that favors allele transmission into the polar body would lead to preferential maternal transmission (Finseth *et al*., 2015; Fishman & Kelly, 2015). Here, since genotype frequencies fulfill the HWE, the driver alleles should outcompete the nondriver alleles in both male and female gametogenesis (Malik, 2005). Many mechanisms can lead to gametic drive in plants, because gametes undergo haploid cell division, a step that does not exist in animals (Finseth, 2023). As a consequence, *c*. 60% of plant genes are expressed in the haploid phase (Chettoor *et al*., 2014; Rutley & Twell, 2015; Klepikova *et al*., 2016), and variants have been identified in genes controlling cell division in gametes of *A. thaliana* (Parker *et al*., 2025).

In addition to segregation distortion at the gametic level, we also detected regions in the genome that experienced biased transmission as a result of the distorted frequency of heterozygotes. One locus on Chromosome 3, for example, was markedly depleted in heterozygotes, despite equal transmission of the two alleles. This locus was close, but distinct from a second locus that decreased the seed production of heterozygotes by 30%. This pattern, which is also known as allelic underdominance, characterizes the genome of a pair of alleles in *A. thaliana* (Smith *et al*., 2011). The causal variant mapped to structural changes in a tandem array of duplicated genes and altered kinase activity (Smith *et al*., 2011). In selfing species and their hybrids, underdominance will not pose a significant threat to the equal transmission of alleles provided that F1 hybrids are viable – as is the case in this study system – because half of the descendants of heterozygote individuals will return to homozygosity.

Allelic underdominance is a form of Dobzhansky–Muller (DM) incompatibility (Dobzhansky, 1936; Muller, 1942). DM incompatibilities arise after alleles are fixed in the diverging parental lineages without being naturally selected to function together. Interactions between alleles segregating at different loci can also cause DM incompatibilities, but, interestingly, no segregation distortions were detected in any major interlocus in this system, nor did any interactive QTLs determine fertility. DM incompatibilities have been studied extensively in many taxa (Bomblies & Weigel, 2007; Masly & Presgraves, 2007; White *et al*., 2012; Schumer *et al*., 2014; Zuellig & Sweigart, 2018; Coughlan & Matute, 2020). Although such incompatibilities are assumed to evolve completely neutrally, they can also result from local adaptation (Alcázar *et al*., 2014).

However, whereas only two loci showed underdominance, six loci showed selection for heterozygote genotypes. This discrepancy indicates that in this system the compensation of deleterious variants in heterozygote regions is more predominant than the emergence of DM incompatibilities (Clo *et al*., 2021). Indeed, six regions in the genome show an excess of heterozygotes. This imbalance indicates that the low effective population sizes of the two species, as well as their relatively recent origin, will have increased the fixation of deleterious alleles faster than they will have fixed new incompatible mutations (Simons & Sella, 2016; Dittberner *et al*., 2022). As recombination and selfing will proceed in further generations after hybridization, high‐fitness individuals are expected to arise that will have purged these variants. The interplay between purging and recombination has been shown to cause heterogeneous rates of introgression along the genome of hybridizing taxa (Schumer *et al*., 2018).

The pattern of past introgression in the *Arabis* system is in fact heterogeneous along the genome (Dittberner *et al*., 2022). Yet, our work shows that the selective removal of deleterious variants is probably not the only force affecting this system today. The massive transmission advantage of the *A. sagittata* alleles on Chromosomes 4 and 7 implies that any allele that is located close to the driver of the distortion will readily introgress into the local *A. nemorensis* population, whether it is deleterious or ecologically relevant. However, linked QTLs of small effect in the vicinity of the distortion will be hard to detect. Other approaches, such as transcriptome analyses, are needed to determine the potential ecological relevance of genetic variation hitchhiking with gametic driver alleles.

The genomics of allele transmission is clearly shaping the consequences of hybridization in this system. Yet, segregation distortions, incompatibilities, and overdominance were detectable on a limited number of loci. Due to their simple genetic basis, the loci are unlikely to block genetic exchanges (Li *et al*., 2022). The genetic architecture of the comprehensive panel of traits quantifying growth rate, growth form, the transition to flowering, plant height, and the production of seeds allowed us to examine the extent to which gene flow at ecologically relevant QTLs was hindered by genomic barriers in this system. Results demonstrated that the parental lineages differ genetically in most traits, with *c*. 48% of the QTLs contributing to this variation; that is, most traits are independent of the loci affecting segregation or fertility in the F2 generations (Table S5).

Hybrid F2 offsprings often exhibited phenotypic values beyond the range of the parental lineages, particularly for plant height, rosette diameter, leaf length, and flowering time. Indeed, although the two parental genotypes did not differ in the timing of flowering, it was for this trait that the largest QTL was detected on Chromosome 8. Our study showed that changes in flowering time in *A. nemorensis* and *A. sagittata* hybrids are not linked to the well‐known genes controlling flowering time, *FLC* or *CO* (Alonso‐Blanco *et al*., 2009; Andrés & Coupland, 2012). Instead, the fine‐mapping of the largest QTL defined a genetic region that contains only one candidate gene for flowering time. This gene, first described as *TFL1* in *A. thaliana*, accelerates flowering by decreasing the number of vegetative side buds in this species (Alvarez *et al*., 1992; Moraes *et al*., 2019). In the perennial species *A. alpina*, it further interacts with the age pathway to prevent juvenile plants from responding to vernalization, thereby controlling the transition out of the juvenile stage, an additional developmental transition of central importance for perennials (Wang *et al*., 2011). Because this flowering time variant is independent of all segregation distortions and fertility alleles, it appears as a potential adaptive trait likely to be reshaped as a result of gene flow. Studies of flowering time provided some of the most remarkable examples of contemporary adaptation to climate change in plants (Hancock *et al*., 2024), and adaptive introgression of flowering time alleles has been documented in several cases (Le Corre *et al*., 2020; Todesco *et al*., 2020; Wang *et al*., 2021). Environmental responses to global climate change include advancing flowering time: many plants induce earlier flowering as a strategy to escape warmer temperatures (e.g. Anderson *et al*., 2012; Siegmund *et al*., 2016; Cai *et al*., 2019; Tun *et al*., 2021).

Which additional, genetically different traits may be adaptive in natural communities remains to be determined. Larger leaves, greater lateral spread, and lower specific leaf area, all of which are variable in the descendants of hybrid individuals in this system, have been associated with competitive advantage in the ephemeral wetland of vernal pool plant communities (Kraft *et al*., 2015). Interestingly, the *A. nemorensis* maternal background increased rosette diameter, especially at early stages, while the *A. sagittata* maternal background increased stem leaf length. Both of these traits can improve light capture, but their relative fitness relevance should depend on the trade‐off between survival and competition (Lundgren & Des Marais, 2020). Backcrossing of *A. nemorensis* pollen on *A. sagittata* mother plants appeared to occur more frequently among hybrids (Dittberner *et al*., 2019, 2022), which would thus promote an increase in spell out stem leaf length. Therefore, genetic and maternal variants may contribute to the emergence of a particularly competitive genotype in the dense plant communities of floodplain meadows. In this context, it is intriguing that signatures of putative selective sweeps in the parental species were found for many of the QTLs determining variation among rosette size. However, recent selection in the parental lineages has not been systematically associated with variable QTLs, so that much of the variation manifested in the F2 generation forms a new base of variants that has not yet been shaped by natural selection.

As the climate changes and land‐use intensifies, the native floodplain habitat is increasingly exposed to drought events as well as flooding, both of which will affect plant survival in natural environments and neither of which could be quantified in this common garden experiment (Colloff *et al*., 2016). *In situ* analyses of the performance of hybrid offspring are therefore needed to shed light on the genetic basis of variation in survival rates. Our study allows us to conclude that there is sufficient genetic diversity in our system for hybridization to generate novelty. However, the prospects of such novelties will depend on how selective pressures act in nature on genetically variable traits within the architecture of allelic transmission.

## Competing interests

None declared.

## Author contributions

NR designed and performed the RAD‐seq and QTL mapping analyses, conducted flowering‐time experiments and analyses, and wrote the manuscript. LM contributed to the whole‐genome sequencing (WGS) analyses and wrote the WGS section of the manuscript. LH assisted with RAD‐seq data analysis. KK assisted with genotype correction. ASK generated the genome annotations. YÖ contributed to phenotypic data collection. TA, CID, SA, RYW and NR generated the genome assemblies. GS and BS contributed to data interpretation. AT and JM conceived and supervised the project, contributed to data interpretation and reviewed and edited the manuscript.

## Disclaimer

The New Phytologist Foundation remains neutral with regard to jurisdictional claims in maps and in any institutional affiliations.

## Supporting information


**Fig. S1** Phenotypic variation in fitness‐related traits in *Arabis* parental species and F1 hybrids.
**Fig. S2** Phenotype distribution in *Arabis* F2 progeny and the effect of cross‐direction.
**Fig. S3** Correlation network of phenotypic traits.
**Fig. S4** Correlation heatmap of phenotypic traits.
**Fig. S5** Synteny and rearrangement plot between *Arabis nemorensis* and *Arabis sagittata* genomes.
**Fig. S6** Correlation between genetic and physical distance of SNPs.
**Fig. S7** Mosaic plot of SNP distribution along the genome in the *Arabis* mapping population.
**Fig. S8** F2 population linkage disequilibrium.
**Fig. S9** Genetic architecture of fertility score.
**Fig. S10** QTLs and LOD score distribution.
**Fig. S11** Distribution of flowering time in *Arabis* F3 hybrids.
**Fig. S12** Sweep detection and QTLs across chromosomes for *Arabis nemorensis* and *Arabis sagittata*.
**Fig. S13** Overlap between selective sweep windows and 10% quantile QTL regions across chromosomes in *Arabis nemorensis* and *Arabis sagittata*.
**Methods S1** Common garden experiment and phenotyping.
**Methods S2** Phenotypic analyses.
**Methods S3** Extraction and RAD‐seq library construction.
**Methods S4** Assembly.
**Methods S5** SNP calling and genetic map construction and library construction in *Arabis* F2 progeny.
**Methods S6** Whole genome resequencing.
**Notes S1** Phenotypic analyses supporting information and codes.
**Notes S2** Genome assembly supporting information and codes.
**Notes S3** RAD‐seq analysis supporting information and codes.
**Notes S4** Genetic map construction supporting information and codes.
**Notes S5** QTL mapping analysis supporting information and codes.
**Notes S6** Sweep detection supporting information and codes.
**Notes S7** Flowering time fine‐mapping analysis supporting information and codes.
**Table S1** Overview of mean flowering time for *Arabis* F3 families.
**Table S2** Genotype and phenotype of *Arabis* F3 families used in the flowering time fine‐mapping experiment.
**Table S3** Results of reciprocal cross‐effect analysis on phenotypic traits.
**Table S4** Genetic map overview.
**Table S5** Summary of detected QTLs across traits and their relationship to fertility and distortion regions in *Arabis* F2 progeny.
Please note: Wiley is not responsible for the content or functionality of any Supporting Information supplied by the authors. Any queries (other than missing material) should be directed to the *New Phytologist* Central Office.

## Data Availability

fastq files of the F2 population, genome assemblies, and annotations are available at the European Nucleotide Archive (ENA) under project no. PRJEB89863. All supplementary figures can be found at Figs S1–S13. All scripts, VCF file, genome annotation and phenotypic datasets used in this study are available on Notes S1–S7.
