# Supplementary material for: Contemporary hybridization among Arabis floodplain species creates opportunities for adaptation
Source: New Phytol. 2025 Dec 10;249(3):1542–57. doi: 10.1111/nph.70779 (PMC12780310; doi:10.1111/nph.70779)
Supplement: Supplementary file 1 — Fig. S1 Phenotypic variation in fitness‐related traits in Arabis parental species and F1 hybrids. Fig. S2 Phenotype distribution in Arabis F2 progeny and the effect of cross‐direction. Fig. S3 Correlation network of phenotypic traits. Fig. S4 Correlation heatmap of phenotypic traits. Fig. S5 Synteny and rearrangement plot between Arabis nemorensis and Arabis sagittata genomes. Fig. S6 Correlation between genetic and physical distance of SNPs. Fig. S7 Mosaic plot of SNP distribution along the genome in the Arabis mapping population. Fig. S8 F2 population linkage disequilibrium. Fig. S9 Genetic architecture of fertility score. Fig. S10 QTLs and LOD score distribution. Fig. S11 Distribution of flowering time in Arabis F3 hybrids. Fig. S12 Sweep detection and QTLs across chromosomes for Arabis nemorensis and Arabis sagittata. Fig. S13 Overlap between selective sweep windows and 10% quantile QTL regions across chromosomes in Arabis nemorensis and Arabis sagittata. Methods S1 Common garden experiment and phenotyping. Methods S2 Phenotypic analyses. Methods S3 Extraction and RAD‐seq library construction. Methods S4 Assembly. Methods S5 SNP calling and genetic map construction and library construction in Arabis F2 progeny. Methods S6 Whole genome resequencing. Notes S1 Phenotypic analyses supporting information and codes. Notes S2 Genome assembly supporting information and codes. Notes S3 RAD‐seq analysis supporting information and codes. Notes S4 Genetic map construction supporting information and codes. Notes S5 QTL mapping analysis supporting information and codes. Notes S6 Sweep detection supporting information and codes. Notes S7 Flowering time fine‐mapping analysis supporting information and codes. Table S1 Overview of mean flowering time for Arabis F3 families. Table S2 Genotype and phenotype of Arabis F3 families used in the flowering time fine‐mapping experiment. Table S3 Results of reciprocal cross‐effect analysis on phenotypic traits. Table S4 Genetic map overvie [file NPH-249-1542-s001.docx]

## *New Phytologist* Supporting Information

Article title: Contemporary hybridization among *Arabis* floodplain species creates opportunities for adaptation

Authors: Neda Rahnamae, Lukas Metzger, Lea Hördemann, Kevin Korfmann, Abdul Saboor Khan, Yasar Özoglan, Craig I. Dent, Samija Amar, Raúl Y. Wijfjes, Tahir Ali, Gregor Schmitz, Benjamin Stich, Aurelien Tellier, Juliette de Meaux

Article acceptance date: 10 October 2025

The following Supporting Information is available for this article:

**Fig. S1** Phenotypic variation in fitness-related traits in *Arabis* parental species and F1 hybrids

**Fig. S2** Phenotype distribution in *Arabis* F2 progeny and the effect of cross-direction

**Fig. S3** Correlation network of phenotypic traits

**Fig. S4** Correlation heatmap of phenotypic traits

**Fig. S5** Synteny and rearrangement plot between *A. nemorensis* and *A. sagittata* genomes

**Fig. S6** Correlation between genetic and physical distance of SNPs

**Fig. S7** Mosaic plot of SNP distribution along the genome in the *Arabis* mapping population

**Fig. S8** F2 population linkage disequilibrium

**Fig. S9** Genetic architecture of fertility score

**Fig. S10** QTLs and LOD score distribution

**Fig. S11** Distribution of flowering time in *Arabis* F3 hybrids

**Fig. S12** Sweep detection and QTLs across chromosomes for *A. nemorensis* and *A. sagittata*

**Fig. S13** Overlap between selective sweep windows and 10% quantile QTL regions across chromosomes in *A. nemorensis* and *A. sagittata*

**Table S1 Overview of mean flowering time for *Arabis* F3 families**

**Table S2 Genotype and phenotype of *Arabis* F3 families used in the flowering time fine-mapping experiment**

**Table S3 Results of reciprocal cross-effect analysis on phenotypic traits**

**Table S4 Genetic map overview**

**Table S5 Summary of detected QTLs across traits and their relationship to fertility and distortion regions in *Arabis* F2 progeny**

**Methods S1** Common Garden Experiment and Phenotyping

**Methods S2** Phenotypic Analyses

**Methods S3** Extraction and RAD-seq Library Construction

**Methods S4** Assembly

**Methods S5** SNP Calling and Genetic Map Construction and Library Construction in Arabis F2 progeny

**Methods S6** Whole Genome Re-sequencing

**Notes S1** Phenotypic analyses supporting information and codes

**Notes S2** Genome assembly supporting information and codes

**Notes S3** RAD-seq analysis supporting information and codes

**Notes S4** Genetic map construction supporting information and codes

**Notes S5** QTL mapping analysis supporting information and codes

**Notes S6** Sweep detection supporting information and codes

**Notes S7** Flowering time fine-mapping analysis supporting information and codes

**Fig. S1 Phenotypic variation in fitness-related traits in *Arabis* parental species and F1 hybrids.** Each point represents the mean value per accession from three populations (described in Dittberner *et al.* 2022), including number of seeds, number of siliques, mean seed weight, silique length; diamonds indicate the median. Letters above data points denote significant differences between groups (*p*<0.05). The parental species (*A. nemorensis* and *A. sagittata*) were reciprocally crossed to produce F1 hybrids. The fitness of F1 was comparable to that of the parental species. Seedlings were grown in the greenhouse of the Experimental Garden of the University of Cologne, and the seeds of the first generation of selfing (F2) were collected.


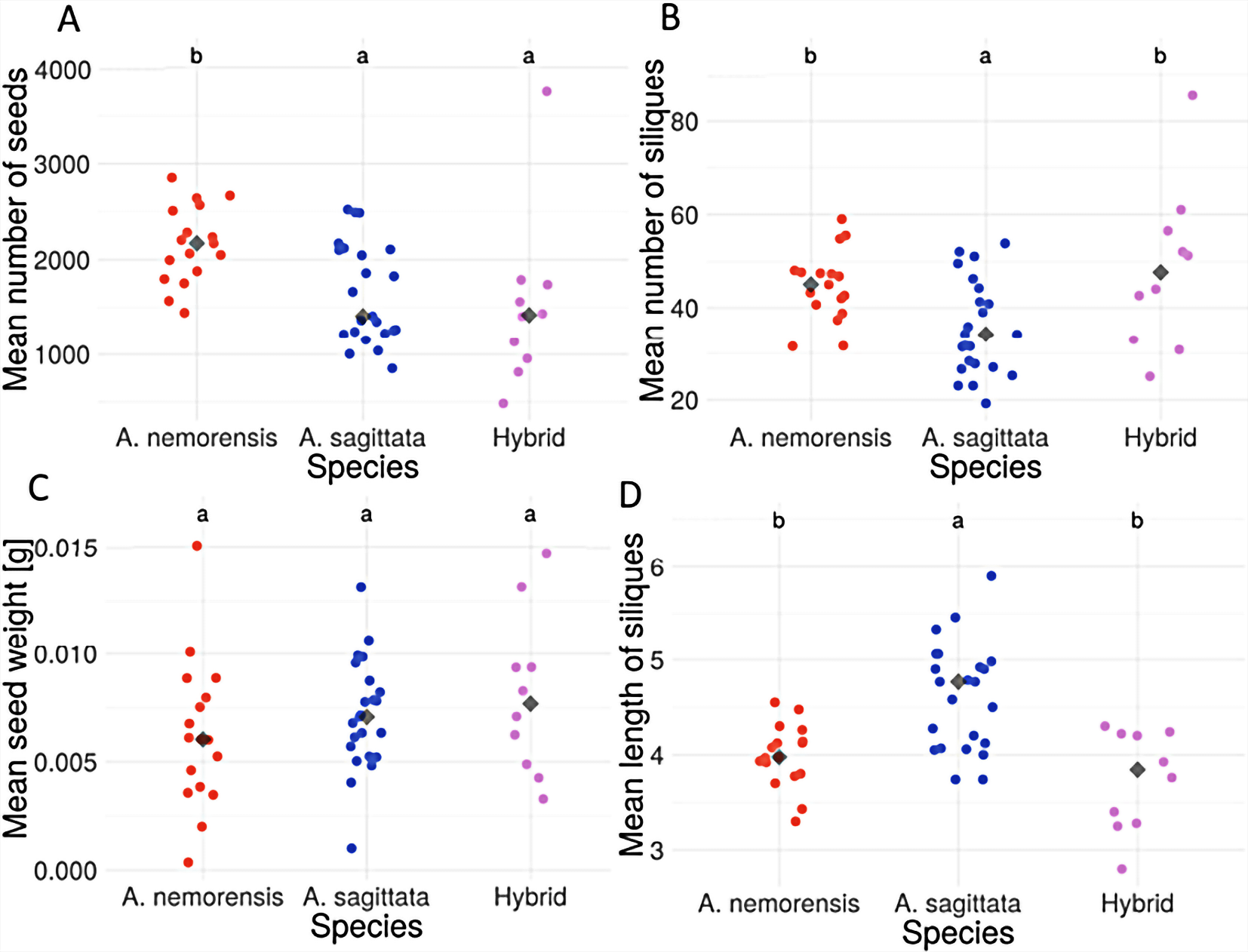


**Fig. S2 Phenotype distribution in *Arabis* F2 progeny and the effect of cross-direction.** Histograms show the distribution of phenotypic traits in 1,193 F2 individuals grown in a common garden, highlighting traits for which the direction of the cross has a significant effect. Colors indicate the maternal species: red represents crosses in which *A. nemorensis* is the female parent (A), and blue represents crosses in which *A. sagittata* is the female parent (B). The p-values for the effect of *A. sagittata* as the female parent on each trait are as follows: Pti: 0.00591, RD1: 0.00008, RD2: 0.01358, RD3: 0.03091, SLL: 0.00003, P.L: 0.01190. When *A. sagittata* is the female parent, petiole length (Pti), stem leaf length (SLL), and the petiole-to-lamina length ratio (P.L) are significantly increased. In contrast, rosette diameter at the first three time points (RD1-RD3) is significantly decreased. For more details, see Table S1.


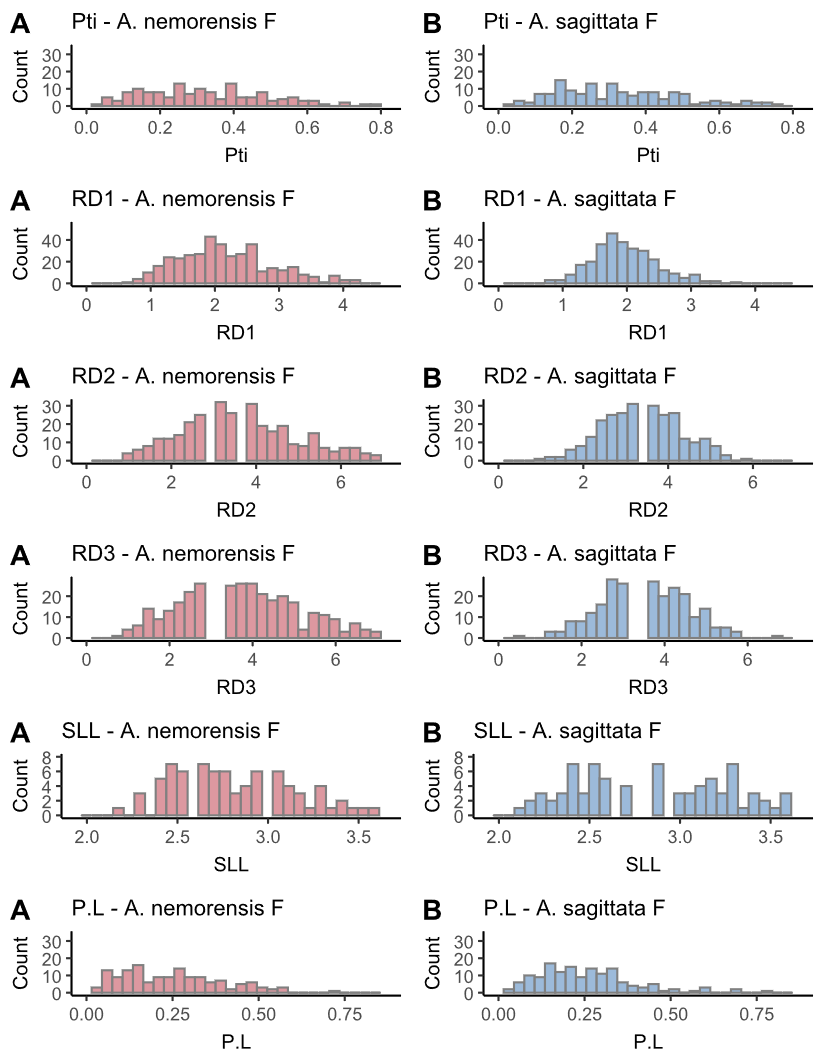


**Fig. S3 Correlation network of phenotypic traits.** The figure above illustrates the relationships among phenotypic traits measured in the common garden experiment in *Arabis* F2 progeny. Spearman’s rank correlation coefficients were conducted in R to quantify pairwise relationships between traits, with residuals from quasi-Poisson models accounting for variation due to experimental blocks and cross-direction. Pairwise correlations were calculated with pairwise deletions for missing data. Edges represent statistically significant correlations (α = 0.05), based on raw *p*-values. The thickness of each edge reflects the strength of the correlation, scaled non-linearly to emphasize stronger relationships: correlations with |r| ≤ 0.5 are drawn with thinner, uniform widths, whereas those with |r| > 0.5 increase in thickness up to a maximum. Partially transparent curved edges improve visual clarity. Positive correlations are shown in green and negative in brown, using a colorblind-friendly palette. The visualization was generated using the ggraph and igraph packages in R, the measured phenotypes include: Days to Bolting (B.T), Days to Flowering (F.T), Fertility Score - Seed Production (W.S), Inflorescence Height (P.H), Lamina Length (Lam), Lamina Length-to-Width Ratio (LLW), Leaf Length (L.L), Leaf Width (L.W), Number of Stem Leaves (N.L), Petal Length (Pet), Petiole Length (Pti), Rosette Diameter at four time points (RD1-4), Side Shoots (Ssh), Stem Height (S.H), Stem Leaf Density (SLD), Stem Leaf Length (SLL), Stem Leaf Width (SLW), Petiole Length-to-Lamina Length Ratio (P.L), and Ground Shoots (Gsh).


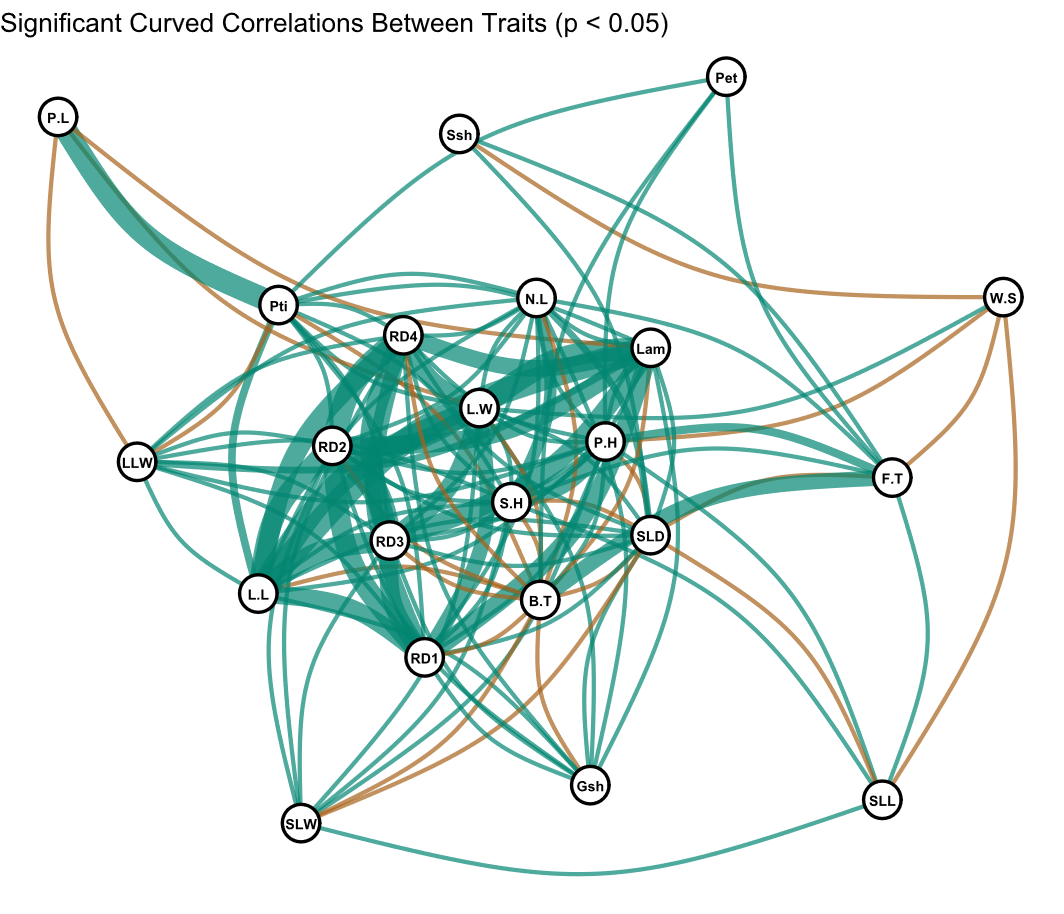


**Fig. S4 Correlation heatmap of phenotypic traits.** This heatmap provides a complementary visualization of the full correlation matrix using Spearman’s rank correlation coefficients measured in *Arabis* F2 progeny. The heatmap is organized hierarchically and displays significant correlations with corresponding labels. A color gradient was applied to distinguish correlation strengths. Positive correlations are shown in green and negative in brown, using a colorblind-friendly palette. Non-significant correlations (α > 0.05) are marked with a cross (×) on their corresponding cells, allowing them to be quickly distinguished from statistically supported relationships. Both visualizations (Figs. S3 and S4) provided complementary insights into the complex association patterns among traits, helping to interpret phenotypic relationships in F2 plants within the common garden experiment. The phenotypes we measured were as follows: Days to Bolting (B.T), Days to Flowering (F.T), Fertility Score - Seed Production (W.S), Inflorescence Height (P.H), Lamina Length (Lam), Lamina Length-to-Width Ratio (LLW), Leaf Length (L.L), Leaf Width (L.W), Number of Stem Leaves (N.L), Petal Length (Pet), Petiole Length (Pti), Rosette Diameter at four time points (RD1-4), Side Shoots (Ssh), Stem Height (S.H), Stem Leaf Density (SLD), Stem Leaf Length (SLL), Stem Leaf Width (SLW), Petiole Length-to-Lamina Length Ratio (P.L), and Ground Shoots (Gsh).


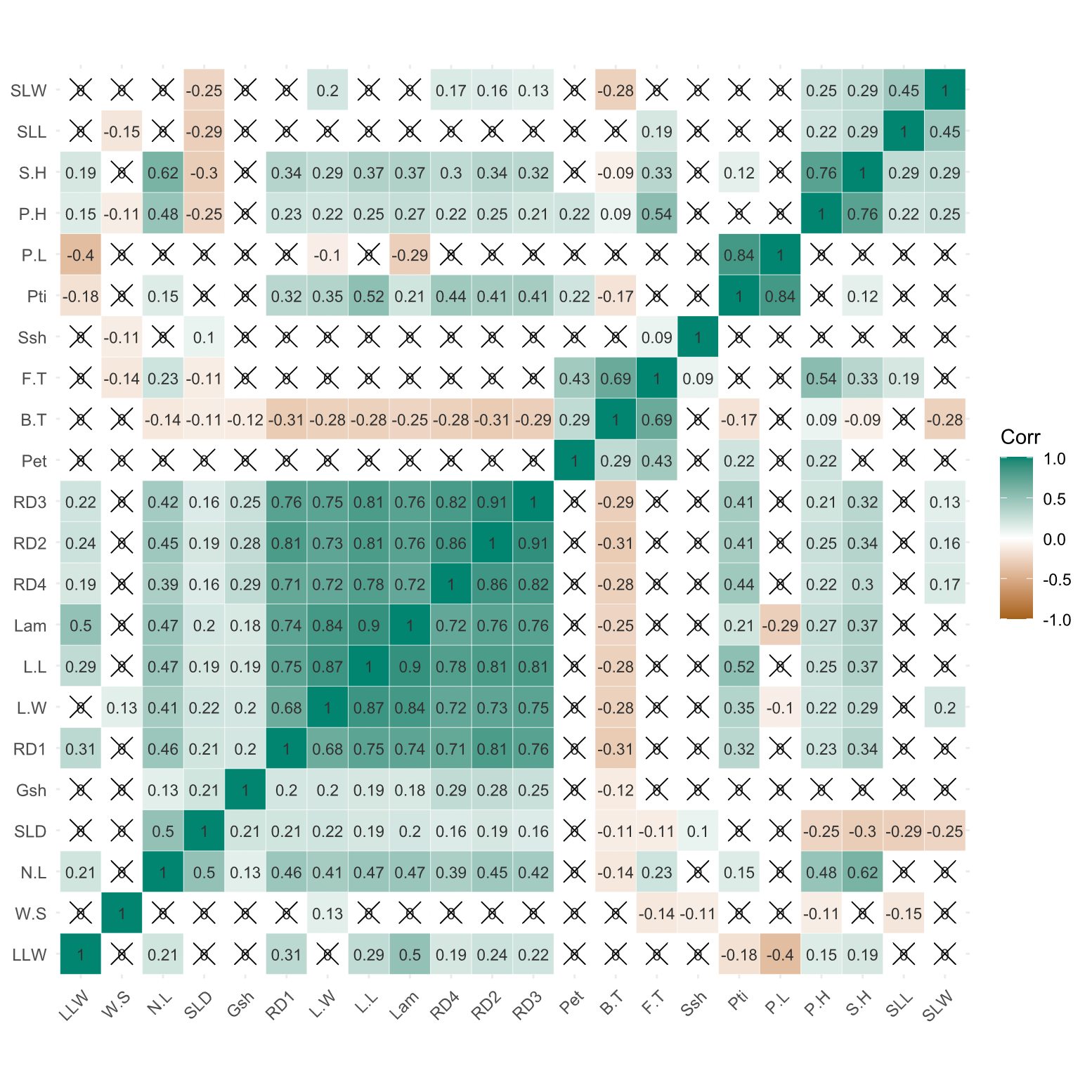


**Fig. S5 Synteny and rearrangement plot between *A. nemorensis* and *A. sagittata* genomes.** The dot plot illustrates synteny and the localization of genomic rearrangements between the final RagTag-generated assemblies of *A. nemorensis* and *A. sagittata*. The x-axis represents scaffolds (1 to 8) of *A. sagittata* (from left to right); the y-axis represents scaffolds (1 to 8) of *A. nemorensis* (from top to bottom). Potential inversions are observed on chromosomes 3, 4, 5, 6, and 7.

**
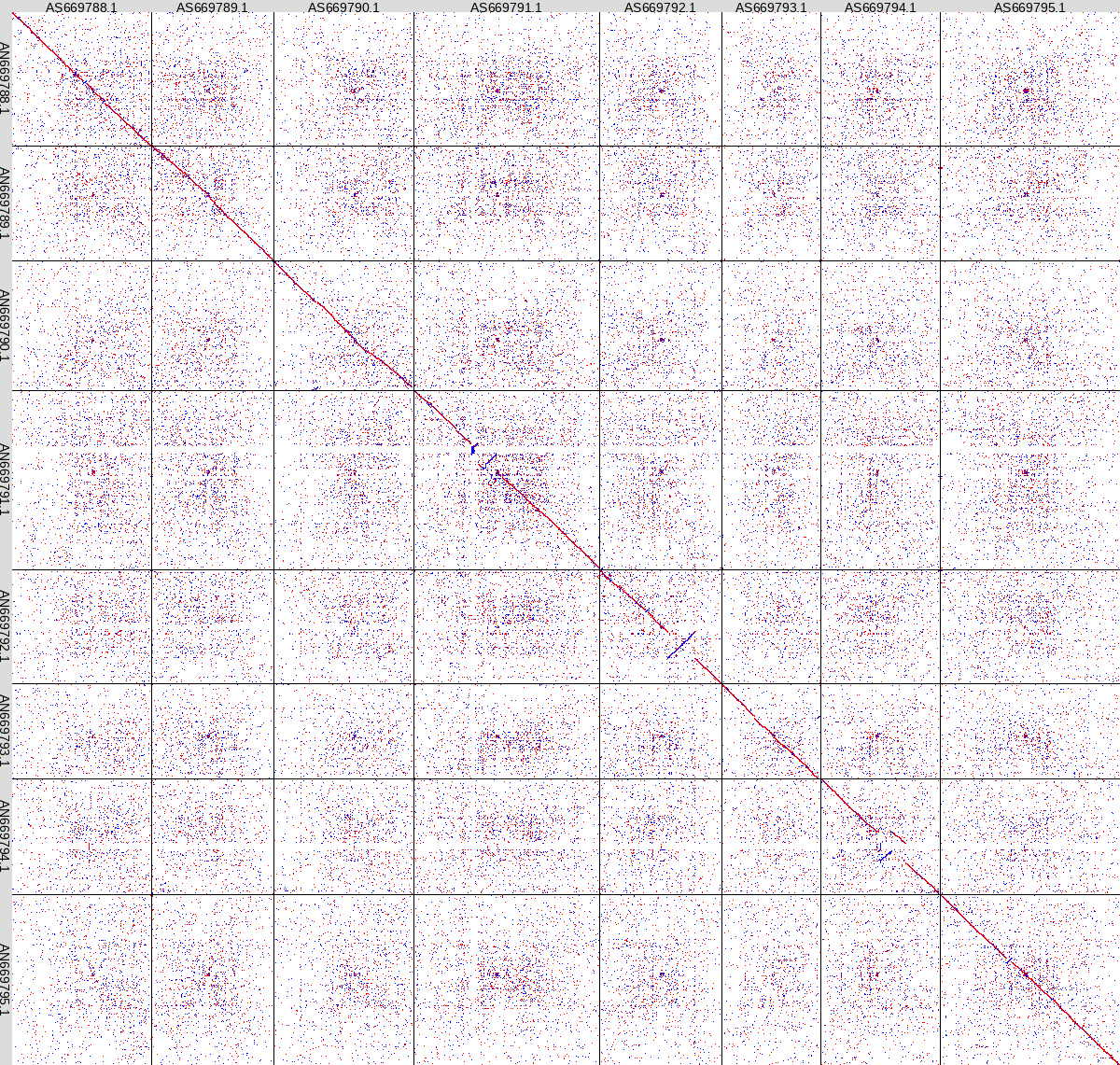
**

**Fig. S6 Correlation between genetic and physical distance of SNPs.** The relationship between genetic and physical distances is shown across the eight linkage groups constructed from 742 *Arabis* F2 individuals genotyped at 2,082 reliable SNP markers. The x-axis represents the physical distance (Mb; Megabase pairs); the y-axis shows the genetic distance (cM; centimorgan) of markers along the chromosomes.


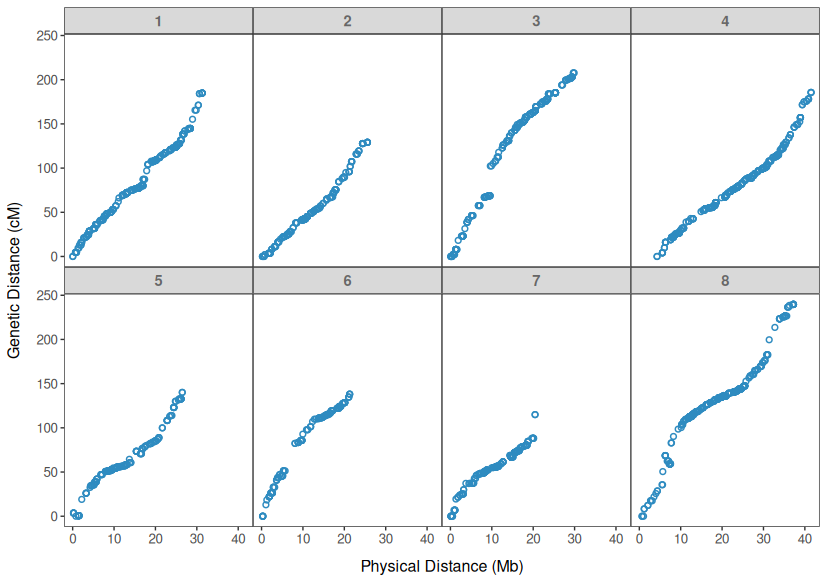


**Fig. S7 Mosaic plot of SNP distribution along the genome in the *Arabis* mapping population.** The plot depicts the distribution of 2,082 SNP markers across the genome for each of the 742 individuals in the mapping population. Different colors represent the genotypes observed at each marker: orange = NN, purple = NS, green = SS, and gray = missing data (N: *A. nemorensis* allele; S: *A. sagittata* allele). The y-axis represents individuals, the x-axis shows the SNP markers distributed across 8 chromosomes. This visualization highlights the recombination breakpoints within the chromosomes of each individual.


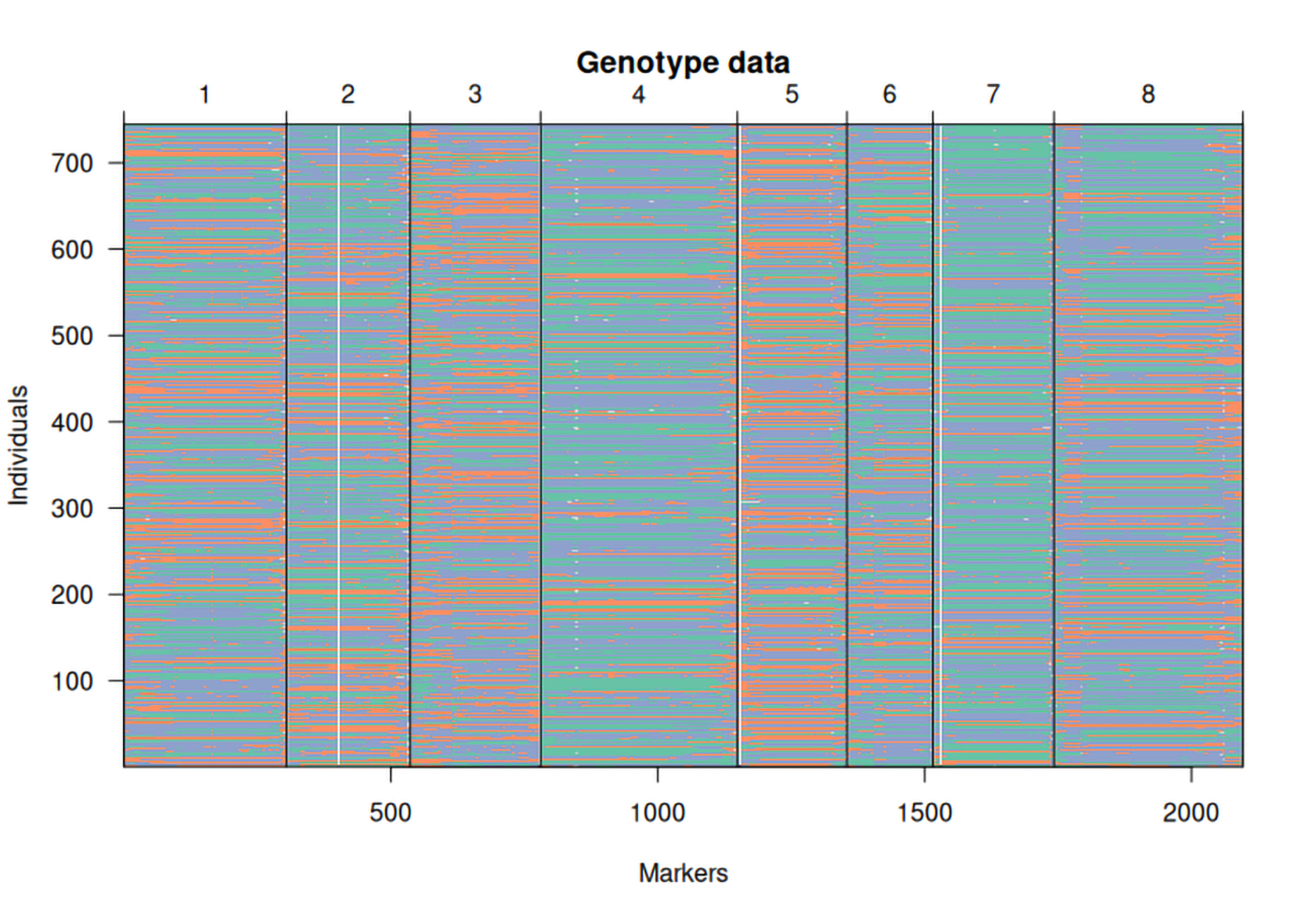


**Fig. S8 F2 population linkage disequilibrium.** Linkage disequilibrium in the F2 population quantified as the correlation coefficient of *A. sagittata* allele counts between each pair of markers. The highest linkage is found among loci on the same chromosome, as a result of which the 8 chromosomes are visible and displayed in order. Significant interchromosomal associations of parental alleles were detected between chromosomes 2 and 5, and chromosomes 3 and 8. The resolution of the map is not sufficient to isolate interacting loci. An anomaly is visible on chromosome 3 around the locus showing a strong depletion in heterozygous individuals.

**Fig. S9 Genetic architecture of fertility score.** (A) The genetic architecture of Fertility Score and segregation distortion in *Arabis* F2 progeny are displayed. The width of the bars represents the strength of the LOD score for each QTL. (B) The effect of the strongest Fertility Score QTL located on chromosome 3 is shown, highlighting its inter-allelic incompatibility.

**
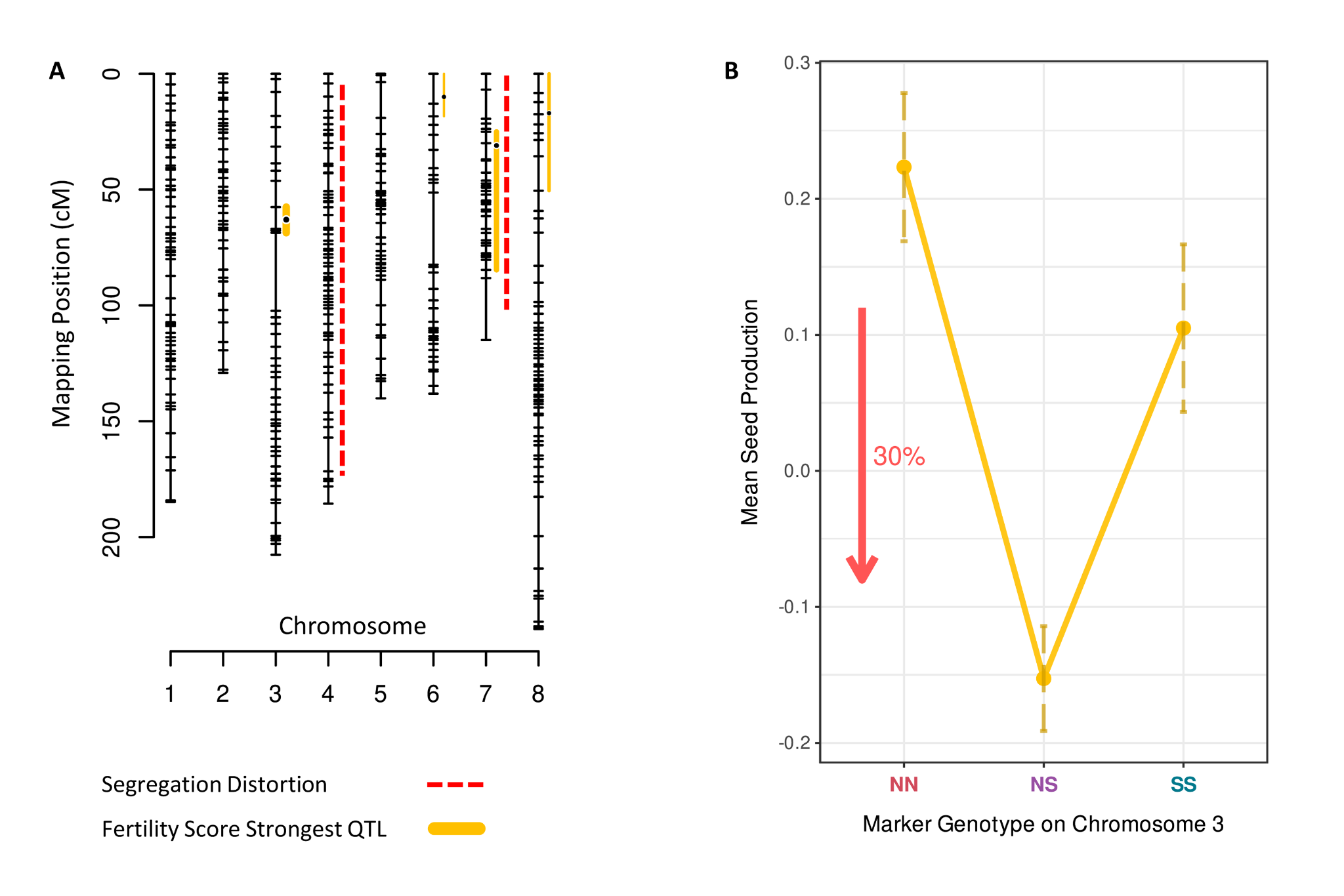
**

**Fig. S10 QTLs and LOD score distribution.** The figure above shows the distribution of ecologically relevant traits in QTLs detected in *Arabis* F2 population LOD scores. Each block represents one QTL and color phenotypes.


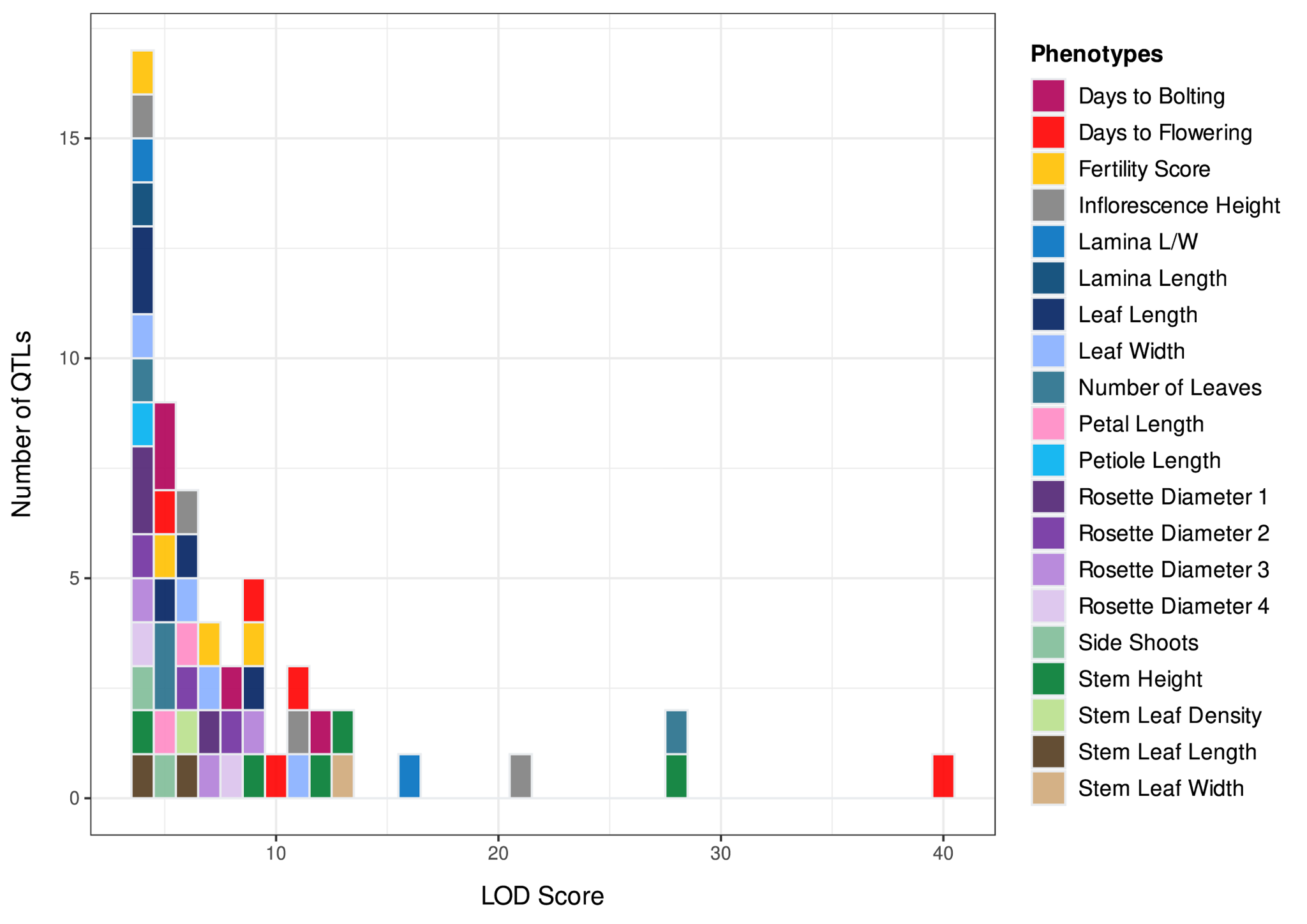


**Fig. S11 Distribution of flowering time in *Arabis* F3 hybrids.** The figure represents the distribution of flowering time among 410 F3 plants across two trials. These plants belong to 15 different genotypic F2 families. Individuals with recombination (full dot) allow the QTL region to be narrowed down. In trial 1, family 825, which had the most replicates, had the latest mean flowering time, 233 days (n=23, SD=4.50). Conversely, family 1,094 flowered earliest and had the lowest variation, averaging 222 days (n=15, SD=1.62). In trial 2, plants generally displayed earlier flowering than plants in trial 1 (Table 5). This may be due to differences in environmental conditions such as temperature or light intensity, despite efforts to maintain consistent settings in both common garden experiments. Family 1,094 had the earliest flowering time, with a mean of 198 days (n=19, SD=5.86), whereas family 885 had the latest flowering time,204 days (n=34, SD=5.43). The lowest variation was observed in family 173, the highest in family 263.


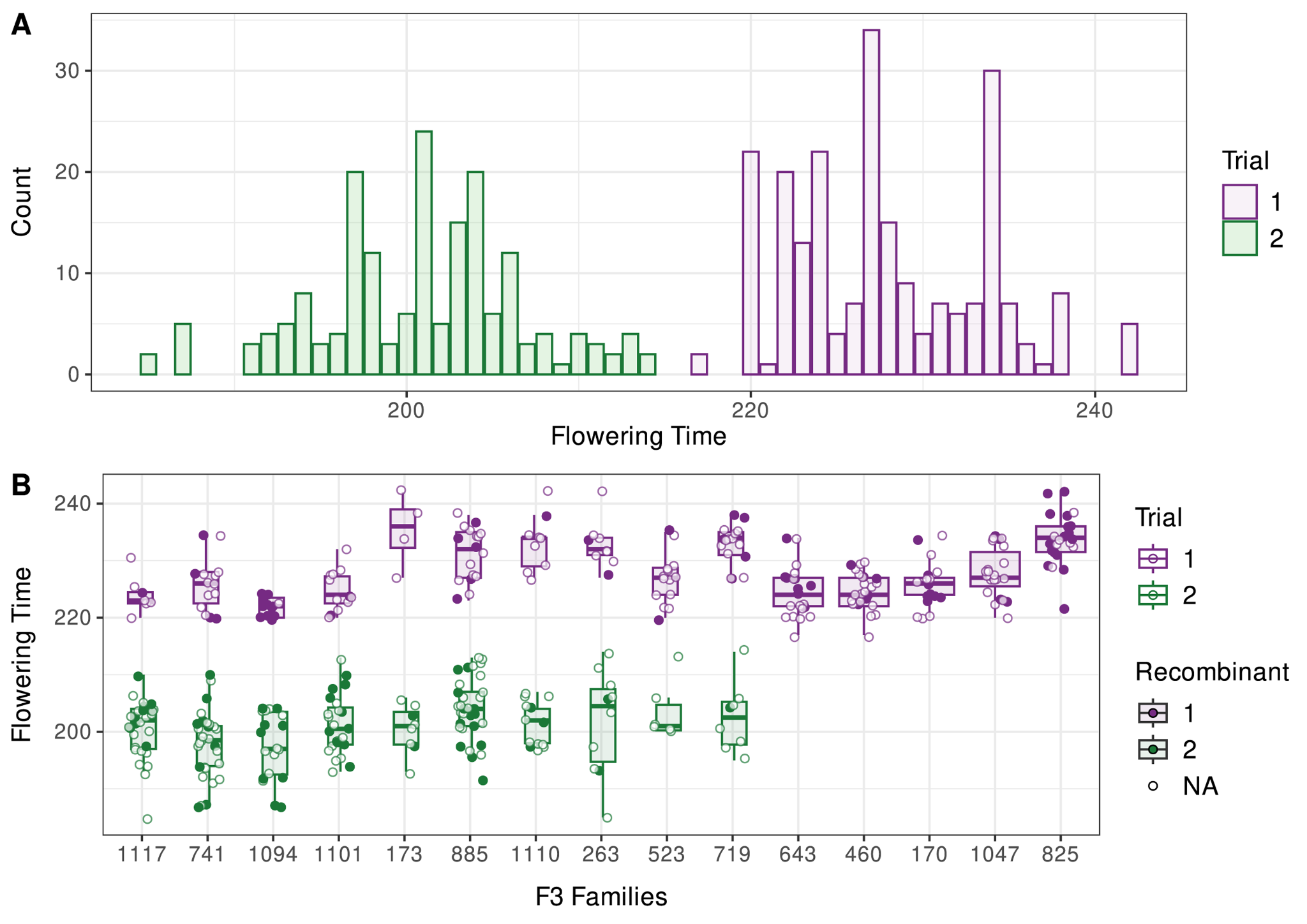


**Fig. S12 Sweep detection and QTLs across chromosomes for *A. nemorensis* and *A. sagittata*.** The plot shows detected selective sweeps (lines) and the positions of all QTL regions (rectangles) for various phenotypic traits, with QTLs categorized by trait type and represented in different colors. Each chromosome is displayed in a separate facet, with likelihood values of sweeps plotted along the y-axis. A dashed horizontal line indicates the likelihood threshold for significant sweep detection. Each QTL bar is adjusted in width and positioned along the y-axis based on its chromosome index, and black dots mark QTL peak positions. The likelihood curves are represented for both species, *A. nemorensis* (red) and *A. sagittata* (blue).


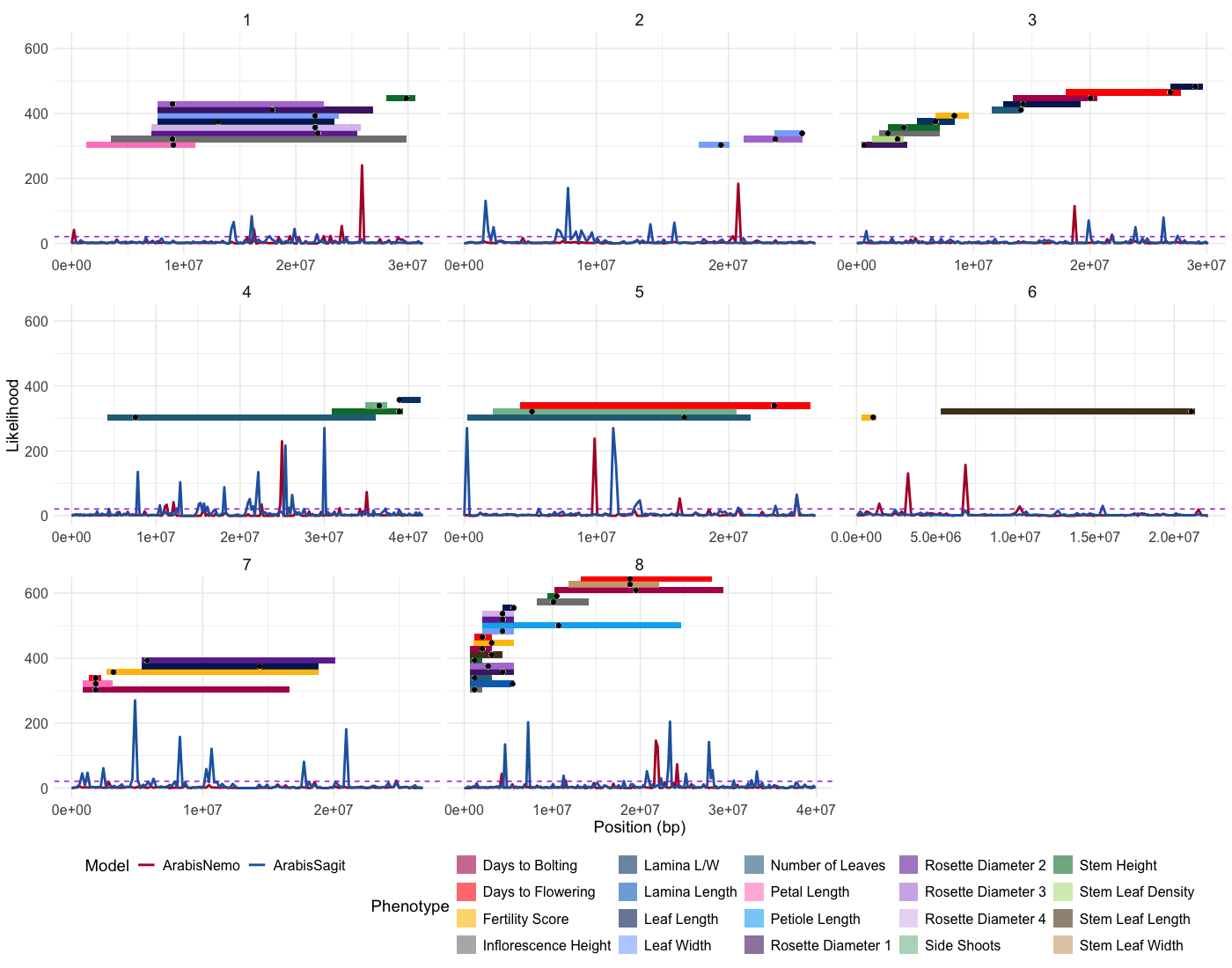


**Fig. S13 Overlap between selective sweep windows and 10% quantile QTL regions across chromosomes in *A. nemorensis* and *A. sagittata*.** The plot shows selective sweeps as horizontal lines, each representing a ±100,000 bp region centered around detected sweep positions. Only overlapping QTLs (with sweeps) are shown as colored horizontal bars, extending to a 10% quantile range around their peak positions. Black vertical lines indicate QTL peak positions within these regions. Different colors denote specific phenotypic traits associated with each QTL; sweeps are colored according to regions found within each species.


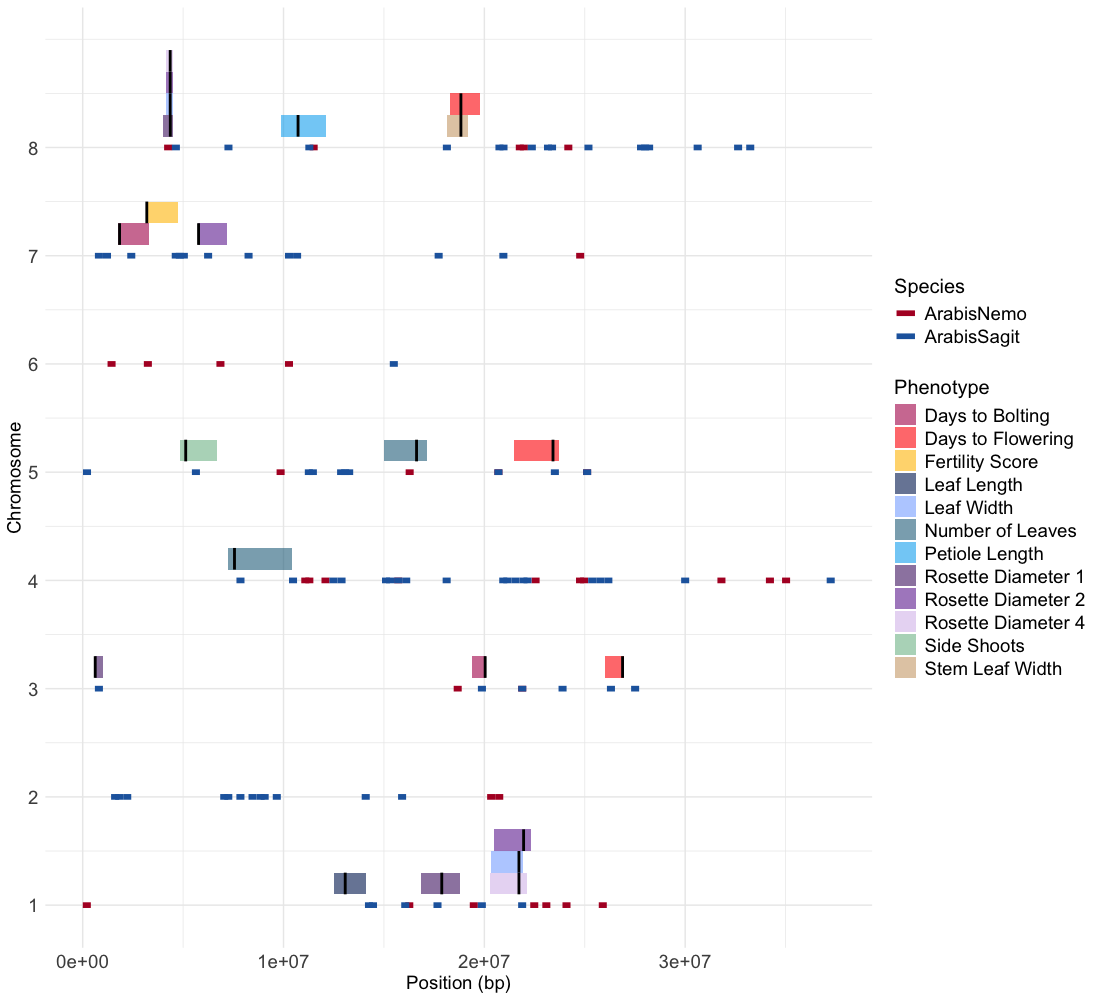


**Notes S1** Phenotypic analyses supporting information and codes: <https://github.com/nedarahnama/Contemporary_hybridization/tree/master/01_phenotypic_analyses>

**Notes S2** Genome assembly supporting information and codes: <https://github.com/nedarahnama/Contemporary_hybridization/tree/master/02_genome_assembly>

**Notes S3** RAD-seq analysis supporting information and codes: <https://github.com/nedarahnama/Contemporary_hybridization/tree/master/03_rad_seq>

**Notes S4** Genetic map construction supporting information and codes: <https://github.com/nedarahnama/Contemporary_hybridization/tree/master/04_genetic_map>

**Notes S5** QTL mapping analysis supporting information and codes: <https://github.com/nedarahnama/Contemporary_hybridization/tree/master/05_qtl_mapping>

**Notes S6** Sweep detection supporting information and codes: <https://github.com/Luker121/OverlapSweepQTL/tree/main>

**Notes S7** Flowering time fine-mapping analysis supporting information and codes: <https://github.com/nedarahnama/Contemporary_hybridization/tree/master/06_fine_mapping>

**Table S1** Overview of mean flowering time for Arabis F3 families. This table provides the mean flowering time for each F3 family across the two trials of the fine-mapping experiment. Trial 2 did not include all 15 families.

| **F3 Family** | **Mean Flowering Time in Trial 1** | **Mean Flowering Time in Trial 2** |
| --- | --- | --- |
| 170 | 226 | *NA* |
| 173 | 235 | 200 |
| 263 | 233 | 202 |
| 460 | 225 | *NA* |
| 523 | 227 | 204 |
| 643 | 224 | *NA* |
| 719 | 233 | 202 |
| 741 | 226 | 198 |
| 825 | 234 | *NA* |
| 885 | 231 | 204 |
| 1047 | 228 | *NA* |
| 1094 | 222 | 198 |
| 1101 | 225 | 201 |
| 1110 | 233 | 201 |
| 1117 | 224 | 201 |

**Table S2** Genotype and phenotype of Arabis F3 families used in the flowering time fine-mapping experiment. The table below shows the genotypes for all flowering time QTLs. Q4 is the strongest QTL which was used for fine mapping. “S” represents A. sagittata homozygous; “N,” A. nemorensis homozygous; and “SN,” heterozygous genotypes. The flowering time observed in the F2 common garden experiment is also listed. Family 173 is not included in this table. These families were selected based on a genetic map constructed using an earlier version of the genome assembly.

| QTL ID | Chr | 170 | 263 | 460 | 523 | 643 | 719 | 741 | 825 | 885 | 1047 | 1094 | 1101 | 1110 | 1117 |
| --- | --- | --- | --- | --- | --- | --- | --- | --- | --- | --- | --- | --- | --- | --- | --- |
| Q1 | 3 | N | S | N | S | N | S | N | S | N | N | N | N | S | N |
| Q2 | 5 | N | S | S | NS | N | N | N | NS | N | N | S | N | NS | NS |
| Q3 | 7 | N | NS | S | N | S | S | NS | S | S | S | NS | NS | NS | NS |
| Q4 | 8 | NS | NS | NS | NS | NS | NS | NS | NS | NS | NS | NS | NS | NS | NS |
| Q5 | 8 | N | S | N | S | N | S | N | S | S | NS | N | N | S | S |
| Flowering time | | 190 | 196 | 187 | 193 | NA | 195 | NA | 195 | 193 | 183 | 181 | 181 | NA | 183 |

**Table S3** Results of reciprocal cross-effect analysis on phenotypic traits. The table summarizes the p-values from the analysis of cross-direction and maternal influence on phenotypic traits in Arabis F2 population. Significant p-values are bold for emphasis.

| **Trait** | **Estimated Effect *A. sagitatta* Female** | ***p-value*** |
| --- | --- | --- |
| Days to Bolting (B.T) | 0.007349 | 0.59110 |
| Days to Flowering (F.T) | 0.008801 | 0.45541 |
| Fertility Score (W.S) | 0.046190 | 0.85410 |
| Inflorescence Height (P.H) | 0.007968 | 0.91211 |
| Lamina Length (Lam) | -0.120190 | 0.16244 |
| Lamina L/W (LLW) | 0.015500 | 0.75859 |
| Leaf Length (L.L) | -0.043710 | 0.60346 |
| Leaf Width (L.W) | -0.128730 | 0.09654 |
| Number of Stem Leaves (N.L) | -0.054995 | 0.46388 |
| Petal Length (Pet) | 0.015750 | 0.67500 |
| **Petiole Length (Pti)** | **0.681090** | **0.00591** |
| **Rosette Diameter 1 (RD1)** | **-0.323510** | **0.00008** |
| **Rosette Diameter 2 (RD2)** | **-0.207410** | **0.01358** |
| **Rosette Diameter 3 (RD3)** | **-0.193682** | **0.03091** |
| Rosette Diameter 4 (RD4) | -0.000311 | 0.99680 |
| Side Shoots (Ssh) | -0.322387 | 0.05804 |
| Stem Height (S.H) | -0.005128 | 0.93638 |
| Stem Leaf Density (SLD) | -0.023045 | 0.73420 |
| **Stem Leaf Length (SLL)** | **0.215120** | **0.00003** |
| Stem Leaf Width (SLW) | 0.066639 | 0.27150 |
| **Petiole L/Lamina L (P.L)** | **0.698930** | **0.01190** |
| Ground Shoots (Gsh) | -0.223140 | 0.47830 |

**Table S4** Genetic map overview. This table summarizes the physical and genetic lengths of each chromosome in Arabis F2 progeny, along with the number of SNP markers. The total number of SNPs included in the map is 2,082.

| **Chromosome** | **Physical Length (Mb)** | **Genetic Length (cM)** | **Number of SNPs** |
| --- | --- | --- | --- |
| 1 | 32 | 185 | 304 |
| 2 | 26 | 130 | 232 |
| 3 | 30 | 208 | 244 |
| 4 | 41 | 186 | 369 |
| 5 | 27 | 140 | 202 |
| 6 | 22 | 138 | 160 |
| 7 | 21 | 115 | 220 |
| 8 | 38 | 240 | 351 |

**Table S5** Summary of detected QTLs across traits and their relationship to fertility and distortion regions in Arabis F2 progeny. This table includes the trait name, chromosome (Chr), QTL peak position (in bp [base pairs] and cM [centimorgan]), and the corresponding LOD score for each detected QTL. The final column (“Independence”) indicates whether each QTL is classified as independent (TRUE) or not independent (FALSE), based on its location and potential overlap with fertility-associated QTLs or known segregation distortion regions (chromosomes 4 and 7). QTLs were considered independent if they were not located on chromosomes 4 or 7 and did not overlap with any fertility QTLs. In total, 48.3% of the QTLs were classified as independent.

| **Trait** | **QTL ID** | **Chr** | **Position (bp)** | **Position (cM)** | **LOD** | **Independence** |
| --- | --- | --- | --- | --- | --- | --- |
| Days to Bolting | Q1 | 3 | 20040893 | 163.00 | 5.15 | TRUE |
| Days to Bolting | Q2 | 7 | 1835566 | 21.49 | 5.36 | FALSE |
| Days to Bolting | Q3 | 8 | 2061023 | 12.24 | 7.79 | FALSE |
| Days to Bolting | Q4 | 8 | 19500950 | 134.21 | 12.40 | TRUE |
| Days to Flowering | Q1 | 3 | 26882840 | 190.00 | 10.00 | TRUE |
| Days to Flowering | Q2 | 5 | 23418119 | 111.00 | 4.69 | TRUE |
| Days to Flowering | Q3 | 7 | 1835566 | 21.49 | 11.23 | FALSE |
| Days to Flowering | Q4 | 8 | 2061023 | 13.00 | 40.45 | FALSE |
| Days to Flowering | Q5 | 8 | 18832553 | 133.00 | 8.91 | TRUE |
| Fertility Score | Q1 | 3 | 8362080 | 63.00 | 8.77 | FALSE |
| Fertility Score | Q2 | 6 | 1052484 | 10.00 | 3.90 | FALSE |
| Fertility Score | Q3 | 7 | 3192272 | 31.00 | 6.66 | FALSE |
| Fertility Score | Q4 | 8 | 3118527 | 17.00 | 5.07 | FALSE |
| Inflorescence Height | Q1 | 1 | 9004320 | 49.89 | 4.17 | TRUE |
| Inflorescence Height | Q2 | 3 | 2664384 | 27.00 | 11.39 | FALSE |
| Inflorescence Height | Q3 | 8 | 1180103 | 6.00 | 20.79 | FALSE |
| Inflorescence Height | Q4 | 8 | 10128705 | 101.00 | 6.38 | TRUE |
| Lamina Length | Q1 | 8 | 5481058 | 36.00 | 4.33 | FALSE |
| Lamina L/W | Q1 | 3 | 6744303 | 61.00 | 15.64 | FALSE |
| Lamina L/W | Q2 | 4 | 38897287 | 163.00 | 4.04 | FALSE |
| Leaf Length | Q1 | 1 | 13071968 | 72.39 | 4.46 | TRUE |
| Leaf Length | Q2 | 3 | 14255522 | 136.22 | 5.21 | TRUE |
| Leaf Length | Q3 | 3 | 29031007 | 201.41 | 5.62 | TRUE |
| Leaf Length | Q4 | 7 | 14342773 | 68.78 | 4.28 | FALSE |
| Leaf Length | Q5 | 8 | 5624776 | 35.00 | 9.43 | FALSE |
| Leaf Width | Q1 | 1 | 21722429 | 116.00 | 3.87 | TRUE |
| Leaf Width | Q2 | 2 | 19435694 | 87.00 | 7.10 | TRUE |
| Leaf Width | Q3 | 2 | 25576894 | 129.00 | 6.34 | TRUE |
| Leaf Width | Q4 | 8 | 4356973 | 32.00 | 10.78 | FALSE |
| Number of Leaves | Q1 | 3 | 14075550 | 130.95 | 4.58 | TRUE |
| Number of Leaves | Q2 | 4 | 7558305 | 18.00 | 4.00 | FALSE |
| Number of Leaves | Q3 | 5 | 16624270 | 70.69 | 5.38 | TRUE |
| Number of Leaves | Q4 | 8 | 1180103 | 7.00 | 28.11 | FALSE |
| Petal Length | Q1 | 1 | 9074627 | 51.00 | 4.86 | TRUE |
| Petal Length | Q2 | 7 | 1835566 | 21.49 | 5.69 | FALSE |
| Petiole Length | Q1 | 8 | 10723493 | 107.50 | 3.81 | FALSE |
| Rosette Diameter 1 | Q1 | 1 | 17879753 | 95.00 | 4.50 | TRUE |
| Rosette Diameter 1 | Q2 | 3 | 623617 | 2.31 | 3.63 | TRUE |
| Rosette Diameter 1 | Q3 | 8 | 4362085 | 28.00 | 6.94 | FALSE |
| Rosette Diameter 2 | Q1 | 1 | 21954996 | 115.00 | 6.16 | TRUE |
| Rosette Diameter 2 | Q2 | 7 | 5769578 | 42.70 | 3.84 | FALSE |
| Rosette Diameter 2 | Q3 | 8 | 4356973 | 31.00 | 7.82 | FALSE |
| Rosette Diameter 3 | Q1 | 1 | 9004320 | 49.89 | 9.39 | TRUE |
| Rosette Diameter 3 | Q2 | 2 | 23541930 | 118.00 | 4.02 | TRUE |
| Rosette Diameter 3 | Q3 | 8 | 2718077 | 18.00 | 7.21 | FALSE |
| Rosette Diameter 4 | Q1 | 1 | 21722429 | 115.29 | 4.33 | TRUE |
| Rosette Diameter 4 | Q2 | 8 | 4356973 | 32.00 | 7.81 | FALSE |
| Side Shoots | Q1 | 4 | 36501835 | 137.73 | 3.86 | FALSE |
| Side Shoots | Q2 | 5 | 5129643 | 35.50 | 4.88 | TRUE |
| Stem Height | Q1 | 1 | 29847516 | 162.00 | 4.37 | TRUE |
| Stem Height | Q2 | 3 | 4035375 | 36.00 | 13.09 | FALSE |
| Stem Height | Q3 | 4 | 38897287 | 160.00 | 8.78 | FALSE |
| Stem Height | Q4 | 8 | 1180103 | 6.00 | 28.31 | FALSE |
| Stem Height | Q5 | 8 | 10506892 | 102.00 | 12.25 | TRUE |
| Stem Leaf Density | Q1 | 3 | 3491752 | 29.00 | 5.76 | TRUE |
| Stem Leaf Length | Q1 | 6 | 21070306 | 134.77 | 5.92 | TRUE |
| Stem Leaf Length | Q2 | 8 | 3118527 | 15.00 | 3.97 | FALSE |
| Stem Leaf Width | Q1 | 8 | 18832576 | 132.55 | 13.23 | TRUE |

**Methods S1** Common Garden Experiment and Phenotyping

To generate the hybrids we crossed sympatric *A. nemorensis* genotype 10 with the *A. sagittata,* genotype 69, collected from the banks of the Rhine River near Mainz in Riedstadt, Hessen, Germany in 2015 and fully sequenced (Dittberner *et al.*, 2019; Dittberner *et al.*, 2022). Because nucleotide diversity within the population is very low (*A. nemorensis* π = 4.37e-5 and *A. sagittata* π = 1.32e-5, synonymous sites, Dittberner *et al.*, 2022), we assume here that differences between these genotypes will predominantly reflect differences between species. Plants were reciprocally crossed to generate F1s, and we noted that their fitness was comparable to that of the parental species (Fig. S1). Seedlings were grown in the greenhouse of the Experimental Garden of the University of Cologne, and the seeds of the first generation of selfing (F2) were harvested. We sowed F2 seeds in trays on 05.10.2019, and after two weeks of vernalization and germination we transplanted seedlings into 7x7cm pots filled with *Topferde* soil (Einheitserde, Sinntal-Altengronau, Germany), placing one seedling per pot. In total, 1,204 individual plants (both hybrids and parental replicates) were distributed across 43 trays, which were put in cold frames to accelerate growth and prevent frost damage from 08.11 to 13.12.2018. On 13.12.2018, we transferred trays to bird-protected cages under seminatural conditions. On 19.11.2018, photographs of each tray were taken using a Canon EOS and rosette sizes were measured using ImageJ. Also, on 19.12.2018, we started harvesting leaves from plants that were big enough (two leaves per plant, ~50mg). Harvesting continued in January, February, and March 2019. Harvested rosette leaves were stored at -80°C for DNA extraction.

Since *A. nemorensis* grows in floodplain meadows, we also tested whether the species differed in tolerance to submergence. A total of 199 F2 individual plants, along with seven *A. nemorensis* and seven *A. sagittata*, were submerged in transparent boxes, each containing 17 L of water for seven weeks in cold frames where plants had been placed 10 days earlier on 03.01.2019. Plants were randomized and distributed across eight boxes, which were kept in cold frames throughout the submergence experiment. After seven weeks of complete submergence, plants were removed from water and left to recover. Then, their survival status was documented. Following a two-week recovery period, we again recorded the survival status of plants, categorizing plants as either dead, survived with new leaf growth, or survived and bolted. During recovery, pots remained in cold frames and were watered as necessary.

Between November 2018 and April 2019, we recorded more than 20 phenotypic traits for each plant, which covered most aspects of plant growth and development. These traits were grouped into five categories: (1) fitness: seed mass from 10 randomly selected siliques or fertility score and survival status after submergence; (2) growth: rosette diameter at four time points (19.11.2018, 08.01.2019, 07.02.2019, 13.03.2019), inflorescence height, and number of side and ground shoots; (3) timing: bolting and flowering times; (4) rosette leaf traits: stem leaf number, leaf length, petiole length, lamina length, lamina length/width ratio, leaf margin type (serrated or smooth), leaf width, and petal length; and (5) stem traits: stem leaf density, stem leaf length and width, and stem height.

**Methods S2** Phenotypic Analyses

To assess phenotypic differences between the two species (*A. nemorensis* and *A. sagittata*), we modelled each trait as a function of species and tray (Trait ~ Species + Tray) using generalized linear models with a quasipoisson error distribution in R. Species contrasts were then estimated using the emmeans package (version 1.11.1), which reports effect sizes on the log scale. We present incidence rate ratio (IRR=exp[estimate]) with 95% confidence intervals and *p*-values. False discovery rate (FDR) correction was applied across traits to control for multiple testing. We used ggplot2 (version 3.5.1; Wickham, 2011) to visualize the distribution of phenotypes for both F2 and parental replicates in a single plot per trait, and to inform our understanding of transgressive segregation within the F2 population (Notes S1).

For assessing genetically based phenotypic correlations between individuals of the F2 population, we employed a generalized linear models of the form Trait ~ Cross + Cross:TrayBlock (shorthand in R as Cross/TrayBlock), with a quasipoisson distribution of errors. Here, Cross indicates the reciprocal cross directions (*A. nemorensis*♀ × *A. sagittata*♂ vs the reciprocal) and TrayBlock represents individual trays, each of which contained plants from only one cross. This structure accounts for cross direction as a fixed effect and tray effects nested within cross direction. Residuals from these models were then extracted for each trait to compute pairwise Spearman correlations among trait value corrected for the experimental design. We used the *corr.test* function from the psych package in R (version 2.4.6.26); pairwise deletions were applied to handle missing data. Trays were not rotated outdoors; positional variance was thus absorbed by the nested tray term. Correlation significance was assessed at α = 0.05. The resulting correlation and *p*-value matrices were used to construct a network in which only significant correlations were retained. A network graph was generated using the igraph (version 2.1.1) and ggraph (version 2.1.1) packages. Each node represents a phenotypic trait, and edges represent statistically significant pairwise correlations. Edge color indicates the sign of the correlation (green for positive, brown for negative), and edge thickness is scaled to the absolute strength of the correlation. Curved edges and partial transparency were used to enhance visual clarity, and node labels were plotted within stylized trait circles. The *ggcorrplot* function from the ggcorrplot package (version 0.1.4.1) was used to visualize the heatmap.

**Methods S3** DNA Extraction and RAD-seq Library Construction

DNA was extracted from *Arabis* F2 leaves stored at -80°C using the Nucleospin® 8 Plant II protocol. Using the restriction-site-associated DNA sequencing (RAD-seq) protocol described in Dittberner *et al.* (2019), we analyzed the genomes of 801 F2 individuals. DNA was quantified with Qubit® 3.0. Fluorometer. For each sample, a total of 250 ng of genomic DNA was digested with the KpnI-HF restriction enzyme (New England Biolabs). Each digested sample was then ligated with one individually barcoded modified Illumina P1 adapter containing 5 bp random nucleotides to PCR duplicates to be removed in the steps that follow. Twenty different barcodes were used, and 52 pools of 20 barcoded individuals each were constructed, with all pools standardized to equal volume and concentration. Libraries were sequenced in four separate runs at the Cologne Center for Genomics (CCG) on the Illumina NovaSeq platform. For 46 pools, sequencing length was 2x100 bp, generating approximately 3 million reads per individual; for the remaining 6 pools, sequencing length was 2x150 bp, generating approximately 6 million reads per individual. These RAD-seq libraries covered, on average, approximately 2% of the 248 Mb genome.

**Methods S4** Genome Assembly and Annotation

In order to improve previous genome assemblies, the DNA of both parental lines (*A. nemorensis* and *A. sagittata*) was sequenced with PacBio HiFi technology and Hi-C data. We used Jellyfish (version 2.3.0; Marçais & Kingsford, 2011) to count k-mers of size 21 in the 2.18 and 1.98 million reads obtained for *A. nemorensis* and *A. sagittata*, respectively. The k-mer histogram generated by Jellyfish was then processed with GenomeScope (version 2.0; Ranallo-Benavidez *et al.*, 2020) to estimate genome size, heterozygosity, and repetitiveness. HiFiAdapterFilt (version 2.0.0; Sim *et al.*, 2022) was used to remove residue PacBio adapter sequences from the HiFi reads. Then, hifiasm was used again to assemble the filtered HiFi reads (version 0.16.1; Cheng *et al.*, 2021; Cheng *et al.*, 2022) with integration of Hi-C data.

Hi-C reads were aligned to the draft contigs using BWA (version 0.7.17; Li & Durbin, 2009), and the resulting alignments were processed with the Juicer pipeline (version 1.6; Durand *et al.*, 2016b) to generate chromatin contact maps. These maps were manually inspected and curated using Juicebox (version 2.20.00; Durand *et al.*, 2016a) to correct misjoins and to anchor and orient contigs into chromosome-scale scaffolds. This integration significantly enhanced the contiguity and accuracy of the assembly.

To scaffold the primary assembly produced by hifiasm, we employed RagTag (version 2.1.0; Alonge *et al.*, 2022), using the *A. alpina* assembly as a reference (Suppl. File 2) to further improve scaffold ordering and correct structural inconsistencies. In addition, a linkage map (see below) allowed us to correct a few minor assembly errors. The final assemblies have been deposited in the European Nucleotide Archive (ENA) and is awaiting an accession number (Project id: PRJEB89863). The ab-initio annotation was generated using the *A. nemorensis* reference genome, which was uploaded to the PlabiPD Helixer platform (https://www.plabipd.de/helixer_main.html). The annotation was performed in “Lineage-Specific” mode with selection of the option “Land Plant”.

**Methods S5** Analysis of RAD-seq Data, SNP Calling, and Construction of Linkage Map in Arabis F2 progeny

RAD-seq read quality was assessed with FastQC (version 0.11.9; Andrews, 2010). PCR duplicates were removed using the clone_filter module in Stacks (version 2.59; Catchen *et al.*, 2013), based on a 5 bp random nucleotide sequence at the end of each adapter. Adapters were trimmed and reads shorter than 60 bp were removed using Cutadapt (version 1.18; Martin, 2011). We demultiplexed samples and filtered out reads with ambiguous barcodes (allowing one mismatch), cut sites, uncalled bases, and low-quality reads (default threshold) using the process_radtags module in Stacks (version 2.59). Reference-based mapping and read-filtering were conducted with BWA (version 0.7.17; Li & Durbin, 2009) using default settings against the *A. nemorensis* reference genome, along with SAMtools (version 1.10; Li *et al.*, 2009) and bash scripts from Rivera-Colón & Catchen (2022) (Suppl. File 3).

We performed variant-calling for 801 individuals using BCFtools mpileup and call (version 1.18; Li *et al.*, 2009) under specific criteria: a base quality score greater than 30, base quality recalculation (-E option), and SNP calling with a significance threshold of *p*-value < 0.05. Genotyped loci were filtered using VCFtools (version 0.1.17; Danecek *et al.*, 2011) to exclude loci with more than 50% missing data across individuals. Only biallelic sites were kept, and indels were removed. Individuals with over 60% missing data were excluded, leaving a working set of 781 individuals for subsequent analysis. Additional filters were applied to site and genotype depth (--min-meanDP 4 --max-meanDP 40 --minDP 5 --maxDP 40), and sites with more than 70% missing data were then removed. Loci spaced less than 100 bp apart were clustered into RAD regions as described by Dittberner *et al.* (2019). Regions with abnormally high or low coverage were excluded based on specific thresholds: mean coverage greater than twice or less than one-third the overall mean, maximum coverage exceeding twice the mean maximum coverage across all regions, or regions shorter than 150 bp. Sites absent from parental lines and ambiguous bases were also removed. Finally, we applied a minor allele frequency filter (--maf 0.25) to the dataset. SNPs (single nucleotide polymorphisms) were extracted using VCFtools (version 0.1.17; Danecek *et al.*, 2011) and custom Python scripts, resulting in a VCF file containing 5,360 SNP markers across 781 individuals (Suppl. File 3).

We applied additional filters to improve the quality of map construction. We removed 39 individuals with missing data exceeding 3,500 loci. Duplicated markers were identified and removed, resulting in 47 markers being discarded. Subsequently, markers with more than 23% missing data were filtered out. To analyze the remaining 2,164 markers, we examined their distribution along the genome, compared segregation distortion patterns with genotype proportion and missingness, and filtered out markers identified as outliers based on heterozygosity proportion and inconsistency with neighboring markers. We removed 68 markers with fractions of heterozygotes lower than 25% or higher than 75%. We also used RepeatMasker (version 4.1.6; Smit *et al.*, 2015) on genome annotations to detect and remove markers within repetitive regions. We used repeatmodeler to identifiy repeat families, an approach that was used in Repeatmasker to mask the repetitive regions in the genome. This final analysis produced a genetic map of 2,082 markers distributed across eight chromosomes for 742 individuals (Notes S4).

To improve the accuracy of genotype calling, particularly for heterozygous loci located in low-coverage regions, a Python script was developed to correct incorrectly called genotypes and impute missing data using a sliding window approach. The 2 Mb window size with a 0.5 Mb step size demonstrated the most effective correction and imputation. No further markers or individuals were excluded after this correction and imputation step. The finalized corrected dataset was then used to construct the genetic map using the ASMap package in the R environment (version 1.0-7; Taylor & Butler, 2017) packages in R (version 4.2.3), applying the Kosambi mapping function in the R/qtl package (version 1.66; Broman *et al.*, 2003) as detailed in Notes S4.

Chromosomal orientation was assessed using the constructed genetic map, identifying inversions on chromosomes 3, 4, 6, and 7 due to assembly issues. These regions were adjusted by inverting physical distances on the genetic map, and the genome assembly was subsequently corrected. See above (Genome Assembly and Annotation) for details on the genome assembly process.

**Methods S6** Analysis of Whole Genome Re-sequencing Data

We used previously published resequencing data for 37 *A. nemorensis* and *A. sagittata* individuals (Dittberner *et al.*, 2022). Additionally, we processed one *A. androsacea* individual used as an outgroup (Dittberner *et al.*, 2022).

Reads were mapped to the new version of the *A. nemorensis* reference genome (see Genome Assembly and Annotation) using BWA mem (version 0.7.17; Li & Durbin, 2009) and default parameters. Read depth and other relevant read alignment quality control metrics were computed using QualiMap v.2.2.1 (Okonechnikov *et al.*, 2016). Average read depth across all 37 samples was 23x. Variant calling was performed using GATK version 3.8 (McKenna *et al.*, 2010) and duplicates were marked using PicardTools. Filtering was based on quality thresholds (DP<20; QD<2; MQ < 42; FS>60; SOR > 4; ReadPosRankSum < -6; MQRankSum < -10.5). SNPs were required to be biallelic, and sites at which more than 30% of samples were heterozygous and more than 20% missing were discarded. We polarized *A. nemorensis* and *A. sagittata* individuals with a custom Python script using *A. androsacea* as an outgroup. We performed phasing using the program shapeit2 (Delaneau *et al.*, 2014), assuming a generation time of 1 year and mutation rate per generation of 7×10-9 and recombination rates based on the genetic map described below. From these steps, our dataset resulted in 12,868,614 SNPs before filtering, from which we extracted 4,455,806. While curating the assembly we discovered that segments on chromosomes 3, 4, 6, and 7 were inverted relative to the genetic map. Rather than remapping the reads, we lifted over the VCFs themselves: for every variant falling inside an inverted block, we mirrored its coordinate (new = start + end – old) and reverse-complemented the REF and ALT alleles. The corrected VCF files were used for all downstream analyses.

## References

**Alonge M, Lebeigle L, Kirsche M, Jenike K, Ou S, Aganezov S, Wang X, Lippman ZB, Schatz MC, Soyk S. 2022.** Automated assembly scaffolding using RagTag elevates a new tomato system for high-throughput genome editing. *Genome Biology* 23.

**Andrews S. 2010.** FastQC: a quality control tool for high throughput sequence data. 2010. *Https://Www.Bioinformatics.Babraham.Ac.Uk/Projects/Fastqc/*.

**Broman KW, Wu H, Sen Ś, Churchill GA. 2003.** R/qtl: QTL mapping in experimental crosses. *Bioinformatics* 19.

**Catchen J, Hohenlohe PA, Bassham S, Amores A, Cresko WA. 2013.** Stacks: An analysis tool set for population genomics. *Molecular Ecology* 22.

**Cheng H, Concepcion GT, Feng X, Zhang H, Li H. 2021.** Haplotype-resolved de novo assembly using phased assembly graphs with hifiasm. *Nature Methods* 18.

**Cheng H, Jarvis ED, Fedrigo O, Koepfli KP, Urban L, Gemmell NJ, Li H. 2022.** Haplotype-resolved assembly of diploid genomes without parental data. *Nature Biotechnology*.

**Danecek P, Auton A, Abecasis G, Albers CA, Banks E, DePristo MA, Handsaker RE, Lunter G, Marth GT, Sherry ST, *et al.* 2011.** The variant call format and VCFtools. *Bioinformatics* 27.

**Delaneau O, Marchini J, McVeanh GA, Donnelly P, Lunter G, Myers S, Gupta-Hinch A, Iqbal Z, Mathieson I, Rimmer A, *et al.* 2014.** Integrating sequence and array data to create an improved 1000 Genomes Project haplotype reference panel. *Nature Communications* 5.

**Dittberner H, Becker C, Jiao WB, Schneeberger K, Hölzel N, Tellier A, de Meaux J. 2019.** Strengths and potential pitfalls of hay transfer for ecological restoration revealed by RAD-seq analysis in floodplain *Arabis* species. *Molecular Ecology* 28.

**Dittberner H, Tellier A, de Meaux J. 2022.** Approximate Bayesian Computation Untangles Signatures of Contemporary and Historical Hybridization between Two Endangered Species. *Molecular Biology and Evolution* 39.

**Li H, Durbin R. 2009.** Fast and accurate short read alignment with Burrows-Wheeler transform. *Bioinformatics* 25.

**Li H, Handsaker B, Wysoker A, Fennell T, Ruan J, Homer N, Marth G, Abecasis G, Durbin R. 2009.** The Sequence Alignment/Map format and SAMtools. *Bioinformatics* 25.

**Marçais G, Kingsford C. 2011.** A fast, lock-free approach for efficient parallel counting of occurrences of k-mers. *Bioinformatics* 27: 764–770.

**Martin M. 2011.** Cutadapt removes adapter sequences from high-throughput sequencing reads. *EMBnet.journal* 17.

**McKenna A, Hanna M, Banks E, Sivachenko A, Cibulskis K, Kernytsky A, Garimella K, Altshuler D, Gabriel S, Daly M, *et al.* 2010.** The genome analysis toolkit: A MapReduce framework for analyzing next-generation DNA sequencing data. *Genome Research* 20.

**Okonechnikov K, Conesa A, García-Alcalde F. 2016.** Qualimap 2: Advanced multi-sample quality control for high-throughput sequencing data. *Bioinformatics* 32.

**Ranallo-Benavidez TR, Jaron KS, Schatz MC. 2020.** GenomeScope 2.0 and Smudgeplot for reference-free profiling of polyploid genomes. *Nature Communications* 11.

**Rivera-Colón AG, Catchen J. 2022.** Population Genomics Analysis with RAD, Reprised: Stacks 2. In: Methods in Molecular Biology.

**Sim SB, Corpuz RL, Simmonds TJ, Geib SM. 2022.** HiFiAdapterFilt, a memory efficient read processing pipeline, prevents occurrence of adapter sequence in PacBio HiFi reads and their negative impacts on genome assembly. *BMC Genomics* 23.

**Smit AFA, Hubley R, Grenn P. 2015.** RepeatMasker Open-4.0. *RepeatMasker Open-4.0.7.*

**Taylor J, Butler D. 2017.** R package ASMap: Efficient genetic linkage map construction and diagnosis. *Journal of Statistical Software* 79.

**Wickham H. 2011.** ggplot2. *Wiley Interdisciplinary Reviews: Computational Statistics* 3: 180–185.
